# Space–time structure and wavevector anisotropy in space plasma turbulence

**DOI:** 10.1007/s41116-017-0010-0

**Published:** 2018-02-21

**Authors:** Yasuhito Narita

**Affiliations:** 1 Space Research Institute, Austrian Academy of Sciences, Schmiedlstrasse 6, 8042 Graz, Austria; 2 Institut für Geophysik und extraterrestrische Physik, Technische Universität Braunschweig, Mendelssohnstrasse 3, 38106 Braunschweig, Germany; 3 Institute of Physics, University of Graz, Universitätsplatz 5, 8010 Graz, Austria

**Keywords:** Dispersion relation, Anisotropy, Solar wind turbulence

## Abstract

Space and astrophysical plasmas often develop into a turbulent state and exhibit nearly random and stochastic motions. While earlier studies emphasize more on understanding the energy spectrum of turbulence in the one-dimensional context (either in the frequency or the wavenumber domain), recent achievements in plasma turbulence studies provide an increasing amount of evidence that plasma turbulence is essentially a spatially and temporally evolving phenomenon. This review presents various models for the space–time structure and anisotropy of the turbulent fields in space plasmas, or equivalently the energy spectra in the wavenumber–frequency domain for the space–time structures and that in the wavevector domain for the anisotropies. The turbulence energy spectra are evaluated in different one-dimensional spectral domains; one speaks of the frequency spectra in the spacecraft observations and the wavenumber spectra in the numerical simulation studies. The notion of the wavenumber–frequency spectrum offers a more comprehensive picture of the turbulent fields, and good models can explain the one-dimensional spectra in the both domains at the same time. To achieve this goal, the Doppler shift, the Doppler broadening, linear-mode dispersion relations, and sideband waves are reviewed. The energy spectra are then extended to the wavevector domain spanning the directions parallel and perpendicular to the large-scale magnetic field. By doing so, the change in the spectral index at different projections onto the one-dimensional spectral domain can be explained in a simpler way.

## Introduction

Plasmas in space and astrophysical systems often develop into a turbulent state. Examples of turbulent plasmas and magnetic fields can be found in the solar system, too, such as solar atmosphere (photospheric convections, formation of complex magnetic network), interplanetary space (solar wind flow and interplanetary magnetic field), and planetary magnetospheres (shock-upstream and shock-downstream regions, magnetotail region). Magnetic field generation or amplification is possible in the solar and planetary interior by turbulent dynamo processes such as the twisting effect on the magnetic field in a turbulent flow (often referred to as the alpha effect for the Sun, and in general, convective effects are at work in planetary interiors). Understanding plasma turbulence has also immediate implications on the problems of coronal heating, acceleration and transport of galactic cosmic ray, and onset of magnetic reconnection process.

Turbulent fluctuations represent nearly random pattern or motion of the flows, and can be found in our daily experience to geophysical scales such as turbulence in the ocean or in the atmosphere. Our modern understanding of turbulence owes a lot to the picture of energy cascade developed by Richardson (1926) and later formulated as a power-law spectrum for a realization of the inertial range by Kolmogorov (1991). Application of the renormalization method to fluid dynamics was also a success. The Lagrangian-History Direct Interaction Approximation (LHDIA) developed by Kraichnan (1965b) is a demonstration that one can derive Kolmogorov’s inertial-range spectrum from Navier–Stokes equation without introducing any adjustable parameter.

There are two pillars in physics of fluid turbulence. One is the scale invariance and the other is isotropy. Both of them are the properties of Navier–Stokes equation in the inviscid limit (zero viscosity limit), e.g., Frisch (1995). These two properties are related to the scaling symmetry and the rotation symmetry in the fluid system. Turbulence occurs whenever a fluid motion satisfies certain conditions, and occurs independently from types of fluid (gas or liquid). Excitation of randomly-oriented and randomly-sized eddies can be recognized in artistic paintings or drawings. A review by Warhaft (2002) gives an intuitive comparison between random-size and similar-size phenomena. Formation of eddies can be confirmed in many experimental setups for turbulence measurements, e.g., turbulent boundary layer experiment (Falco 1977).

Not all fluid motions develop into turbulence. Transition from a laminar to a turbulent flow can conveniently be characterized by evaluating the Reynolds number,

1 $$\begin{aligned} \mathrm{Re} = \frac{UL}{\nu } . \end{aligned}$$

Here *U* is the characteristic flow speed in the system, *L* the characteristic length scale, and $\nu$ the viscosity of the medium. Laminar flows are representative for low Reynolds numbers (below unity or of the order of unity), while turbulent flows occur at high Reynolds numbers (Frisch 1995). Due to the vast length scale *L* and the small viscosity $\nu$, the plasmas in the solar system and astrophysical systems are characterized by very large Reynolds numbers. The values of the Reynolds number are estimated to be of the order $\mathrm{Re} \sim 10^4$ ($L=10\,\mathrm{cm}$, $U=100\,\mathrm{cm}\,\mathrm{s}^{-1}$) if we walk in the air on the ground and $\mathrm{Re} \sim 10^6$ ($L=10^2\,\mathrm{cm}$, $U=10^3\,\mathrm{cm}\,\mathrm{s}^{-1}$) for a motor vehicle. In geophysical and astrophysical systems both the length scale and the flow speed are large, hence the Reynolds number is also large. Naive estimate gives the Reynolds number of the order of $10^8$, $10^{10}$, and $10^{11}$, in the outer core of the Earth, the solar convection zone, and the galaxy, respectively. It is worth noting here that the estimation of the Reynolds number in astrophysical flows is not trivial, since the medium is in a plasma state. The magnetic Reynolds number and the anisotropy imposed by the large-scale magnetic field need to be considered. See, e.g., Chapter 7.3 in Battaner (1996).

The energy spectrum of turbulence derived by Kolmogorov (1991) is a landmark in the turbulence research. Its derivation is based on the picture that physical processes of turbulence are divided into three distinct ranges: the energy injection range on the largest spatial scales, the inertial range which is regarded as ubiquitous or common in turbulence on the intermediate scales, and the dissipation range on the smallest scales where the diffusive or friction process dominates the flow dynamics and the energy is converted from the kinetic energy into the thermal one. The Kolmogorov spectrum requires also a restoring of isotropy on the inertial-range scales, even if large scales are anisotropic. In contrast, isotropy is considered as broken by the presence of the large-scale magnetic field in space and astrophysical plasmas. Anisotropy appears on various levels of dynamics, ranging from individual particle motions (Lorentz force and Coulomb force) to wave propagations (magnetohydrodynamic waves, for example). In the fluid turbulence treatment, the Reynolds number determines how much the scales are separated from the injection range to the dissipation range. Kolmogorov (1991) also found that the one-dimensional inertial-range spectrum exhibits a power-law as

2 $$\begin{aligned} E^\mathrm{(1D)}(k) = C_\mathrm{K} \epsilon ^{2/3} k^{-5/3} \,, \end{aligned}$$

where the power-law indices for the dissipation rate $\epsilon$ and the wavenumber *k* are determined by the phenomenological model. The coefficient $C_\mathrm{K}$ is called the Kolmogorov constant which needs to be determined by other theoretical treatments, experiments, or simulations, It is, however, interesting to note that the power-law indices can also be derived by dimensional analysis alone. The fluctuation energy (per unit mass) is the squared amplitude and its spectrum has the dimension of energy per wavenumber, $[E] = L^3T^{-2}$. The wavenumber has the dimension $[k] = L^{-1}$. The energy transfer rate (which is regarded as the same as the dissipation rate) has the dimension $[\epsilon ] = L^2T^{-3}$ (energy per time). Formation of the power-law with the slope $-5/3$ in the energy spectrum has extensively been studied and confirmed in various experiments (Saddoughi and Veeravalli 1994).

There are two unique properties in the astrophysical plasmas that make studies of turbulence particularly challenging. The first effect is the coupling with electromagnetic fields. Plasmas represent an ionized gas, and are electrically conducting. Gas dynamics and electromagnetism must be coupled to each other in dynamics. While the inertial range of fluid turbulence essentially represents splitting of eddies toward smaller spatial scales, that of plasma turbulence may represent the energy transport mediated by electromagnetic waves (in addition to eddies). The second effect is the collisionless nature of astrophysical plasmas. The number density is so low (or plasmas are so dilute) that the particle mean free path reaches the same order of the system size. Binary collisions of particles are rare, and energy dissipation in plasma turbulence cannot be explained merely by a diffusion process. Instead, the energy dissipation needs to be explained by wave–particle interactions, i.e., by exchanging energy mediated by the wave electric field.

Power-law formation in the energy spectrum has repeatedly been confirmed since the earliest era of spacecraft measurements in the solar wind. Furthermore, the power-law index is often close to that of the inertial range, $-\,5/3$ (Coleman 1968; Matthaeus and Goldstein 1982; Marsch and Tu 1990; Podesta et al. 2007; Petrosyan et al. 2010). Solar wind serves as a natural laboratory of astrophysical plasma turbulence. Its magnetohydrodynamic picture has been reviewed by Schwenn and Marsch (1991), Tu and Marsch (1995), and Biskamp (2003). Comprehensive review by Bruno and Carbone (2013) presents recent progress in solar wind turbulence research both observationally and theoretically. Physical conditions of plasmas and magnetic fields have been measured by a number of both single and multiple spacecraft missions in situ in interplanetary space. Energy spectra for the magnetic field fluctuations and the flow velocity fluctuations in the solar wind exhibit a power-law in the frequency domain. The power-law index is in the range between $-\,1.5$ and $-\,1.7$. In the higher frequency range (at 0.1–1 Hz and higher in the spacecraft frame) the magnetic energy spectrum shows a break and the fluctuation energy decays more quickly or more steeply than the power-law (Leamon et al. 1998). The energy spectrum has also been studied using the electric field measurement. The electric field spectrum exhibits a power-law with the slope close to that of Kolmogorov’s inertial range at lower frequencies, while the spectrum becomes flatter at higher frequencies (Bale et al. 2005). The ratio of the electric field to the magnetic field is a measure of the wave phase speed (assuming that there is one plane wave at each frequency in the measurement). The phase speed is nearly flow speed at lower frequencies, and increases at higher frequencies. The difference in the spectral slopes at higher frequencies is a sign of dispersive waves. Power-law spectrum for the magnetic field fluctuations extends even to higher frequencies in the range from 1 to 100 Hz in the spacecraft frame (Alexandrova et al. 2009; Sahraoui et al. 2009).

**Fig. 1:**
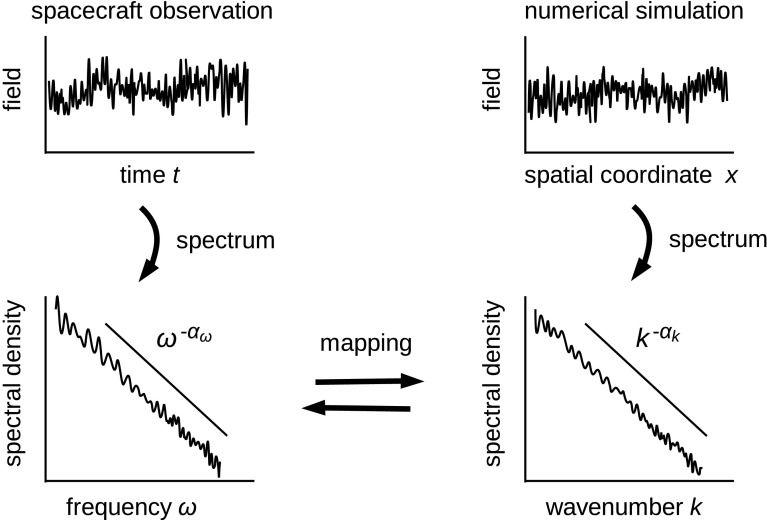
Mapping of the one-dimensional energy spectrum between the frequency domain for the spacecraft observation and the wavenumber domain for the numerical simulation

It is worth mentioning that the one-dimensional energy spectra are typically derived in the spacecraft-frame frequency domain in the observations, while the spectra from numerical simulations (using a magnetohydrodynamic code, a hybrid plasma code, or a particle-in-cell code) are often estimated in the wavenumber domain as illustrated in Fig. 1. Spacecraft data are obtained as time series data, and the Fourier transform of the time series data are obtained in the frequency domain. Simulation data are often stored at snapshots over the spatial coordinates at discrete time steps. The Fourier transform of the simulation data in the spatial coordinates are obtained in the wavenumber or wavevector domain. Both the spectrum in the frequency domain and that in the wavenumber domain exhibit a power law. That is, the frequency spectrum shows a power law $E(\omega ) \propto \omega ^{-\alpha _{\omega }}$ and the wavenumber spectrum also shows a power law $E(k) \propto k^{-\alpha _{k}}$. Moreover, the spectral indices $\alpha _{\omega }$ and $\alpha _{k}$ are often the same or sufficiently close to each other. One may then introduce Taylor’s frozen-in flow assumption (Taylor 1938) and interpret the frequencies as the streamwise wavenumbers, for example, a spectral index of $-\,5/3$. How can we explain this fact? In the presence of finite wave propagations, however, the mapping between the frequency spectra and the wavenumber spectra is no longer unique. The frequency spectra and the wavenumber spectra need to be understood as different projections of the energy spectrum in a higher dimensional sense spanning both the wavenumbers and the frequencies.

Another interesting observation is the possibility that the spectral index can be different between the wavenumber domain and the frequency domain. Different frames are possible for the frequency measurement. In the Eulerian frame, turbulent fluctuations are measured at a spatially fixed point. In the Lagrangian frame, the fluctuations are measured along with a particle in the flow. Phenomenological models address that the one-dimensional energy spectrum in the frequency domain is characterized by a power-law in the both frames but the slope is different between the two frames (Tennekes 1975),

3 $$\begin{aligned} E(\omega )\propto & {} \omega ^{-5/3} \, \, (\mathrm{Eulerian}) \end{aligned}$$

4 $$\begin{aligned} E(\omega )\propto & {} \omega ^{-2} \, \, (\mathrm{Lagrangian}) \,. \end{aligned}$$

Here, the Lagrangian spectrum can be derived from Kolmogorov’s inertial range spectrum by equating the fluctuation energy as $\varDelta k E(k) = \varDelta \omega E(\omega )$ and applying the inertial-range scaling of the velocity fluctuation $V \sim (\epsilon \ell )^{1/3}$ to the Doppler-broadening frequencies as $\varDelta \omega \sim \omega \sim k V \sim \epsilon ^{1/3} k^{2/3}$ (Inoue 1951; Corrsin 1963; Tennekes and Lumley 1972).

**Fig. 2:**
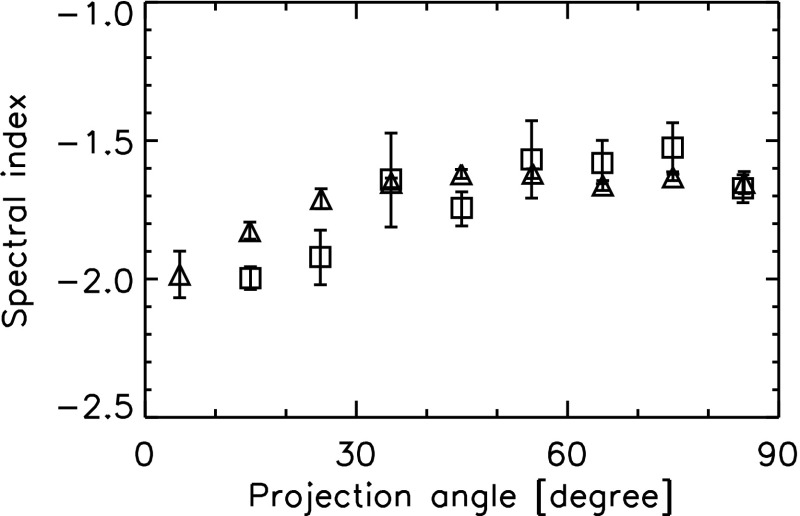
Spectral index of the one-dimensional energy spectra in the spacecraft-frame frequency domain for turbulent magnetic field fluctuations in the solar wind as a function of the projection angles of the flow direction onto the mean magnetic field: measurements by Horbury et al. (2008) (triangles) and Osman and Horbury (2009) (squares)

The existence of the large-scale magnetic field imposes a special direction in plasma dynamics, and in fact, a growing amount of evidence for anisotropy has been presented in various studies of plasma turbulence. Unlike fluid turbulence, the axis of eddies or vortices cannot be oriented randomly in space. Also, electromagnetic waves have preferred propagation directions, depending on their modes. The existence of anisotropy in plasma turbulence has been presented in the studies of solar wind turbulence (Matthaeus et al. 1990; Dasso et al. 2005; Chen et al. 2012), cosmic ray transport (Bieber et al. 1994, 1996), and numerical simulations using different schemes for plasma dynamics such as magnetohydrodynamic treatment (Matthaeus et al. 1996; Matthaeus and Ghosh 1999), ion kinetic or hybrid treatment (Valentini et al. 2010; Verscharen et al. 2012; Comişel et al. 2013), gyro-kinetic treatment (Howes et al. 2011), and full-particle treatment (Saito et al. 2008; Gary et al. 2012; Chang et al. 2013). It is encouraging that using single spacecraft measurements an anisotropic energy spectrum in the three-dimensional wavevector domain was determined by measuring the time correlations, mapping them onto the spatial domain, and then Fourier transforming the spatial correlations onto the wavevector domain (Carbone et al. 1995). Of course, several assumptions have to be made such as vanishing cross helicity (symmetry under reverting the parallel component of the wavevector, $k_\Vert \rightarrow -k_\Vert$) and vanishing magnetic helicity (symmetry under reverting the direction of the wavevector, $\mathbf {k} \rightarrow -\mathbf {k}$), but the estimated spectrum exhibits extended structures (anisotropy) parallel and perpendicular directions to the mean magnetic field. Recent multi-spacecraft measurements in the solar wind provide an evidence for axial asymmetry in the directions around the large-scale magnetic field (Turner et al. 2011). The existence of anisotropy can be found even in the analysis of single spacecraft data in the solar wind. The slope of the energy spectrum (in the frequency domain) is dependent on directions or angles from the large-scale magnetic field (Fig. 2). The slope is close to $-\,5/3$ when the flow and the large-scale field are nearly perpendicular to each other, and close to $-\,2$ when the two directions are nearly parallel or anti-parallel (Osman and Horbury 2009). Another evidence for anisotropy was given by the studies of galactic cosmic ray diffusion (Bieber et al. 1994) to explain the mean free path of particles in a turbulent field.

In this review, we extend the concept of the energy spectrum to higher dimensions. Turbulence is essentially a spatio-temporal phenomenon as pointed out, for example, by Carbone et al. (2011) and Servidio et al. (2011). One-dimensional energy spectrum should be interpreted as an integration or a projection of the energy spectrum in the wavenumber–frequency domain onto the respective one-dimensional domain. For example, integration over the wavenumbers should give the frequency spectrum,

5 $$\begin{aligned} E(\omega ) = \int \mathrm{d}k E(k,\omega ) \end{aligned}$$

and vice versa for the wavenumber spectrum. The two-dimensional energy spectrum $E(k,\omega )$ is related to the space–time correlation by Fourier transform. The random sweeping hypothesis proposed by Kraichnan (1964) provides a convenient tool to visualize the two-dimensional spectrum. Likewise, the anisotropy of turbulent fluctuations is interpreted in the wavevector domain spanning the parallel and the perpendicular components to the large-scale magnetic field.

## Space–time structure

### Hydrodynamic picture

An illustrative and practical model of the wavenumber–frequency spectra (the energy spectra as a function of the wavevectors and the frequencies) is the random sweeping model. The setup comes from the ideal convection problem for three distinct flow velocity fields composed of the mean flow velocity $\mathbf {U}_0$ (assumed to be constant), large-scale variation of flow velocity $\delta {U}$, and small-scale turbulent field $\mathbf {u}$ (assumed to be fully developed). The total flow velocity field is modeled as $\mathbf {U}_0 + \delta \mathbf {U} + \mathbf {u}$, and the Navier–Stokes equation is reduced to an ideal convection equation,

6 $$\begin{aligned} \left( \frac{\partial }{\partial t} + (\mathbf {U}_0 + \delta {U})\cdot \nabla \right) \mathbf {u} = 0 \end{aligned}$$

The wavenumber–frequency spectra can analytically be obtained from Eq. (6) by assuming that the large-scale flow velocity is isotropic and follows the Gaussian statistics (i.e., negligible intermittency effect on larger spatial scales) and Fourier transforming from the spatial domain into the wavevector domain. The small-scale flow velocity is expressed from the convection equation (Eq. 6) in the wavenumber-time domain as

7 $$\begin{aligned} \mathbf {u}(\mathbf {k}, t) = \exp \left[ -\mathrm {i} \mathbf {k} \cdot \left( \mathbf {U}_0 + \delta \mathbf {U} \right) t \right] \, \mathbf {u}(\mathbf {k}, 0) \end{aligned}$$

The energy spectrum for Eq. (7) is obtained as

8 $$\begin{aligned} E(\mathbf {k}, \varDelta t)= & {} E(\mathbf {k}) \left\langle \exp \left[ -\mathrm {i} \mathbf {k} \cdot (\mathbf {U}_0 + \delta \mathbf {U}) \varDelta t \right] \right\rangle \end{aligned}$$

9 $$\begin{aligned}= & {} E(\mathbf {k}) \exp \left[ -\mathrm {i} \mathbf {k} \cdot \mathbf {U}_0 \varDelta t - \frac{1}{6} \langle (\delta U)^2 k^2 (\varDelta t)^2 \rangle \right] , \end{aligned}$$

where the isotropic Gaussian distribution for $\delta U$ is used on the operation of ensemble averaging (denoted by the angular bracket $\langle \cdots \rangle )$. The energy spectrum in the wavenumber–frequency domain is obtained by Fourier transforming from the time domain into the frequency domain. Detailed calculations are shown in Wilczek and Narita (2012). The spectrum is a product of the spectrum in the wavevector domain $E(\mathbf {k})$ and the Gaussian frequency distribution at the given wavevector $F(\omega ,\mathbf {k})$ as:

10 $$\begin{aligned} E(\mathbf {k}, \omega ) = \frac{E(\mathbf {k})}{\sqrt{2\pi k^2 (\delta U)^2}} \exp \left[ -\frac{(\omega - \mathbf {k} \cdot \mathbf {U}_0 )^2}{2 k^2 (\delta U)^2} \right] . \end{aligned}$$

Here, the frequency is measured in the Eulerian frame, i.e., an observer standing in the flow. The Gaussian frequency distribution is centered at the Doppler shift $\omega = \mathbf {k}\cdot \mathbf {U}_0$ and has a Doppler broadening of $\sigma = k \delta U$, where $k = |\mathbf {k}|$ and $\delta U = |\delta \mathbf {U}|$. The Doppler broadening represents a frequency spread around the Doppler shift and is characterized by the standard deviation of the distribution. The large-scale variation of the flow velocity is referred to as the sweeping velocity.

The random sweeping model (Eq. 10) has several important properties in order to understand the space–time structure of turbulent fields in the lowest order sense.

1. The Gaussian frequency distribution $F(\omega , \mathbf {k})$ reduces to the Dirac delta function in the limit of vanishing sweeping velocity, $\delta U \rightarrow 0$:
  11 $$\begin{aligned} F(\mathbf {k}, \omega )= & {} \frac{1}{\sqrt{2\pi k^2 (\delta U)^2}} \exp \left[ -\frac{(\omega - \mathbf {k} \cdot \mathbf {U}_0 )^2}{2 k^2 (\delta U)^2} \right] \end{aligned}$$
  12 $$\begin{aligned}\rightarrow & {} \delta (\omega - \mathbf {k}\cdot \mathbf {U}_0) (\delta U \rightarrow 0) \end{aligned}$$
  such that the streamwise wavenumber–frequency spectrum (in the direction of the mean flow) reduces to
  13 $$\begin{aligned} E(k_\mathrm{flow}, \omega ) = E(k_\mathrm{flow}) \delta (\omega - k_\mathrm{flow}\cdot U_0) . \end{aligned}$$
  Equation (13) is nothing other than Taylor’s frozen-in flow hypothesis (Taylor 1938) in the spectral domain, a one-to-one projection or mapping of the frequencies onto the streamwise wavenumbers using the mean flow speed $U_0$.
2. For an infinitely long inertial-range spectrum in the one-dimensional wavenumber domain, $E(k) \propto k^{-\alpha }$, the spectral index $-\alpha$ of the one-dimensional spectrum is invariant between the frequency domain and the wavenumber domain regardless the choice of the mean flow speed $U_0$ and the large-scale flow speed variation $\delta U$. The frequency spectrum exhibits the same power-law as that in the wavenumber domain with a difference from the wavenumber spectrum only in the coefficient. The energy spectra in the wavenumber domain and the frequency domain are
  14 $$\begin{aligned} E(k)= & {} C_\mathrm{K} \epsilon ^{2/3} k^{-5/3} \end{aligned}$$
  15 $$\begin{aligned} E(\omega )= & {} C(U_0,\delta U) C_\mathrm{K} \epsilon ^{2/3} |\omega |^{-5/3} \,, \end{aligned}$$
  where the coefficient on the frequency spectrum *C*(*U*, *V*) is given by
  16 $$\begin{aligned} C(U_0,\delta U) = \int _0^\infty \mathrm{d}\gamma \frac{\gamma ^{2/3}}{4U_0} \left[ \mathrm{erf}\left( \frac{\gamma + U_0}{\sqrt{2}\, \delta U} \right) - \mathrm{erf}\left( \frac{\gamma - U_0}{\sqrt{2}\, \delta U} \right) \right] . \end{aligned}$$
  The symbol $C_\mathrm{K}$ denotes the Kolmogorov constant, and $\epsilon$ the energy dissipation rate.
3. The Lagrangian-frame frequency spectrum can also be obtained when using the Richardson–Kolmogorov scaling $\delta U \sim (\ell \epsilon )^{1/3}$:
  17 $$\begin{aligned} E(\omega ) = C_\mathrm{L} \epsilon \omega ^{-2} . \end{aligned}$$

**Fig. 3:**
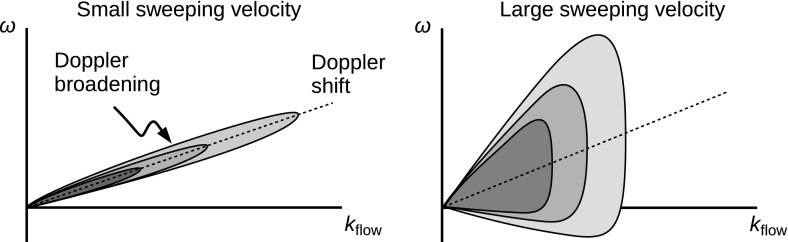
Different realizations of energy spectra for the random sweeping model of hydrodynamic turbulence in the two-dimensional spectral domain spanning the Eulerian frequencies $\omega$ (standing in a flow) and the streamwise wavenumbers $k_\mathrm{flow}$. Doppler shift and broadening over frequencies are associated with the mean flow speed and the large-scale flow speed variation, respectively

The energy spectrum for the random sweeping model is graphically displayed in Fig. 3 under two different conditions of the sweeping velocity $\delta U$ with respect to the mean flow velocity $U_0$. In the case of a small sweeping velocity (left panel), the mapping from the frequencies onto the wavenumbers is, though not exact, well justified because the most of the fluctuation energy is stored along the Doppler shift $\omega = k_\mathrm{flow} U_0$. In other words, the Doppler broadening must be sufficiently small compared to the Doppler broadening for Taylor’s hypothesis to be valid. In fact, the validity of Taylor’s hypothesis can be quantitatively estimated by incorporating the random sweeping model. We define the validity of Taylor’s hypothesis by evaluating the amount of the fluctuation energy being mapped from the streamwise wavenumbers onto the frequencies along the Doppler shift within a frequency bin $\varDelta \omega$. The fluctuation energy in the frequency bin at a given wavenumber is estimated as

18 $$\begin{aligned} I = 2 \int _{k_\mathrm{flow} U_0}^{k_\mathrm{flow} U_0 + \varDelta \omega } \, \mathrm {d}\omega \, \frac{ 1 }{ \sqrt{ 2\pi k_\mathrm{flow}^2 (\delta U)^2 } } \, \exp \left[ -\frac{ (\omega - k_\mathrm{flow}U_0 )^2 }{ 2 k_\mathrm {flow}^2 (\delta U)^2 } \right] . \end{aligned}$$

Here, the factor 2 comes from the symmetry with respect to the Doppler shift $k_\mathrm{flow} U_0$ to the higher frequency $k_\mathrm{flow} U_0 + \varDelta \omega$ and to the lower frequency $k_\mathrm{flow} U_0 - \varDelta \omega$. The use of Taylor’s hypothesis is well justified if the validity parameter *I* is close to unity, $I \simeq 1$. The validity parameter *I* can be expressed by the error function $\mathrm {erf}(x)$ (Narita 2017a) as

19 $$\begin{aligned} I= & {} \mathrm{erf}(\varDelta \tau ) \end{aligned}$$

20 $$\begin{aligned}= & {} \frac{2}{\sqrt{\pi }} \int _{0}^{\varDelta \tau } \, \mathrm{d}\tau \, \exp \left[ -\tau ^2 \right] , \end{aligned}$$

where the variables $\tau$ and $\varDelta \tau$ are introduced as

21 $$\begin{aligned} \tau= & {} \frac{1}{\sqrt{2}} \left( \frac{ \omega }{ k_\mathrm{flow} \delta U } - \frac{U_0}{\delta U} \right) \end{aligned}$$

22 $$\begin{aligned} \varDelta \tau= & {} \frac{1}{\sqrt{2}} \left( \frac{ k_\mathrm{flow} U_0 + \varDelta \omega }{ k_\mathrm{flow} \delta U } - \frac{ U_0 }{ \delta U } \right) \end{aligned}$$

23 $$\begin{aligned}\simeq & {} \frac{1}{\sqrt{2}} \frac{U_0}{\delta U} \frac{\varDelta \omega }{\omega } . \end{aligned}$$

Thus, Taylor’s hypothesis is more valid (the parameter *I* is close to unity) at larger $\varDelta \tau$, i.e., when the ratio of the large-scale flow fluctuation to the mean flow velocity is sufficiently small. In the case of a large sweeping velocity, the fluctuation energy is spread over a wide range of frequencies and is no longer constrained to the Doppler shift. Under a sufficiently large sweeping velocity, the energy may spread over both the positive and negative frequencies, e.g., temporarily returning or backward flow motion due to the large-scale flow variation.

### Magnetohydrodynamic picture

#### Is turbulence strong or weak?

Turbulence serves as a source of energy for the small-scale fluctuations, while serving as a sink of energy for the large-scale structures, e.g., the Reynolds stress tensor in the mean-field dynamics. One speaks of turbulence being “strong” such that the fluctuating field influence the mean field and the large-scale structure becomes deformed or even destroyed by the fluctuating field, and turbulence being “weak” such that the turbulent fluctuations are mostly composed of linear-mode waves.

In the strong turbulence treatment, nonlinearities are so effective that fluctuation amplitudes alter the mean magnetic field, making the mean field inhomogeneous. Both nonlinearities and inhomogeneities need to be taken into account to describe strong turbulence. Various models and concepts have been developed in order to treat strong turbulence theoretically, e.g., mixing length, eddy viscosity, Alfvén time, *k*-$\epsilon$ model (Biskamp 2003). Applications of the strong turbulence approach to solar wind turbulence are found in Yokoi (2006); Yokoi and Hamba (2007); Yokoi et al. (2008).

The weak turbulence theory is considered as valid when the fluctuation amplitudes are small enough, smaller than the mean field (magnetic field and flow velocity) on various spatial scales of interest (Tsytovich 1970; Akhiezer et al. 1975). Fluctuation amplitudes become increasingly smaller to the mean field on smaller length scales. If the fluctuation amplitude is not any more negligible, the dispersion relation becomes deformed by the finite amplitude and the frequencies depend not only on the wavenumbers but also on the amplitudes as

24 $$\begin{aligned} \omega = \omega _0(\mathbf {k}) + \left. \frac{ \partial \omega }{\partial (A^2) } \right| _{\omega _0} + \mathcal {O}(A^4) . \end{aligned}$$

See Chapter 11.2 in Treumann and Baumjohann (1997). Hereafter, we limit ourselves to the weak turbulence picture such that the space–time behavior of the turbulent fluctuations is well described in the wavevector–frequency domain.

#### Wave approach

The Doppler shift has a form of linear dispersion relation. If the turbulent medium has not only a mean flow but also large-scale linear mode waves such as Alfvén waves in a magneto-fluid, the energy spectrum exhibits a Doppler-shifted dispersion relation. Furthermore, if there are multiple wave modes or dispersion relations, the spectrum splits into the respective dispersion relations.

**Fig. 4:**
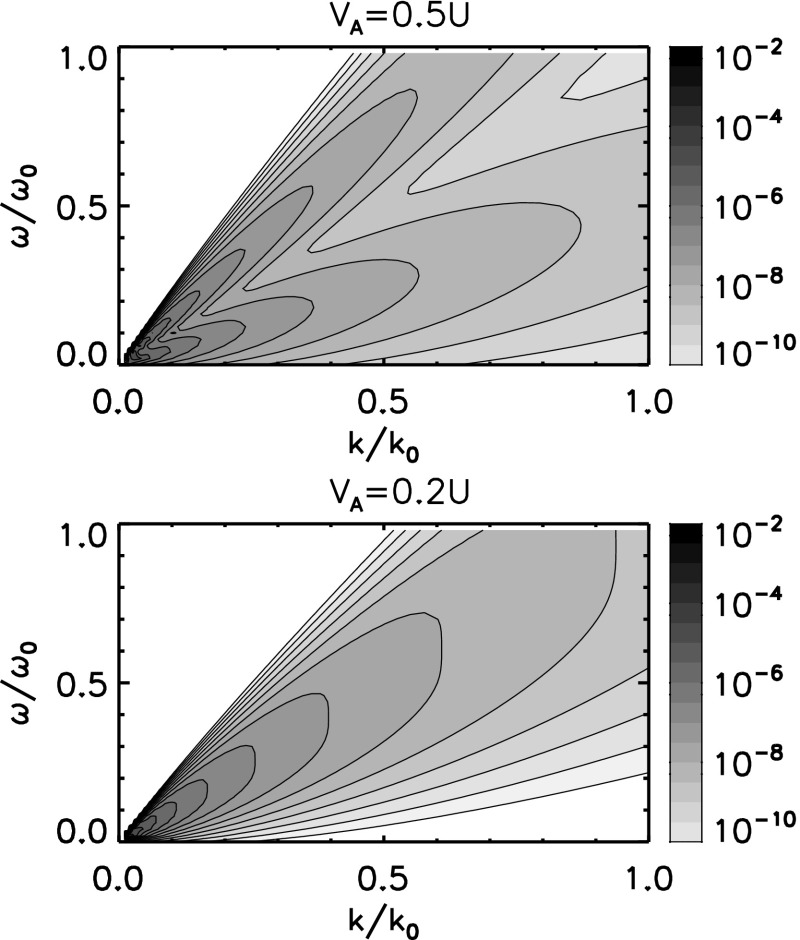
Different realizations of the energy spectra for the random sweeping model of magnetohydrodynamic turbulence in the same spectral domain as that in Fig. 3. The Doppler shift splits into the forward and backward propagating Alfvén waves (with respect to the mean flow direction). Image reproduced with permission from Narita (2017a), copyright by the author

Examples of the energy spectra are displayed in Fig. 4 for a small-scale turbulent field (either the flow velocity field or the magnetic field) swept by the mean flow, the large-scale Alfvén waves propagating both forward and backward to the mean magnetic field, and the random sweeping field respective to the Alfvén waves. The dispersion relations for the forward and backward propagating Alfvén waves in a mean flow are

25 $$\begin{aligned} \omega _+= & {} \mathbf {k}\cdot \mathbf {U}_0 + \mathbf {k} \cdot \mathbf {V}_\mathrm{A} \end{aligned}$$

26 $$\begin{aligned}= & {} k_\mathrm{flow} U_0 + k_\mathrm{flow} V_\mathrm{A} \frac{\cos \theta _\mathrm{kB}}{\cos \theta _\mathrm{kU}} \end{aligned}$$

27 $$\begin{aligned} \omega _+= & {} \mathbf {k}\cdot \mathbf {U}_0 - \mathbf {k} \cdot \mathbf {V}_\mathrm{A} \end{aligned}$$

28 $$\begin{aligned}= & {} k_\mathrm{flow} U_0 - k_\mathrm{flow} V_\mathrm{A} \frac{\cos \theta _\mathrm{kB}}{\cos \theta _\mathrm{kU}} \,, \end{aligned}$$

where $\omega _+$ and $\omega _-$ are the Eulerian frequencies of the forward and backward propagating Alfvén waves, respectively, $\mathbf {V}_\mathrm{A}$ the Alfvén speed, $\theta _\mathrm{kU}$ the angle between the wavevector and the mean flow, and $\theta _\mathrm{kB}$ the angle between the wavevector and the mean magnetic field. The factor $\cos \theta _\mathrm{kB}/\cos \theta _\mathrm{kU}$ is set to unity in Fig. 4 for the sake of simplicity. If the Alfvén speed is sufficiently large (e.g., $V_\mathrm{A} = 0.5 U_0$ in the top panel), the energy spectrum shows two distinct or split dispersion relations, the Doppler shifted Alfvén waves in the forward and backward to the mean flow. In the Alfvén speed is small (e.g., $V_\mathrm{A} = 0.2 U_0$ in the bottom panel), the spectral splitting cannot be resolved but appears as a single peak line or dispersion relation with an enhanced broadening.

The energy spectrum is linear to each wave component in the random sweeping treatment. For counter-propagating Alfvén waves, the spectrum in the wavenumber–frequency domain is obtained from the MHD (magnetohydrodynamic) equations as:

29 $$\begin{aligned} E(\mathbf {k},\omega )= & {} \left( F^{(+)}(\mathbf {k},\omega ) + F^{(-)}(\mathbf {k},\omega ) \right) E(\mathbf {k}) \end{aligned}$$

30 $$\begin{aligned} F^{(\pm )}(\mathbf {k},\omega )= & {} \frac{ 1 }{ \sqrt{2\pi (\sigma ^{(\pm )})^2} } \exp \left[ - \frac{ \left( \omega - \mathbf {k}\cdot (\mathbf {U}_0 \pm \mathbf {V}_\mathrm{A}) \right) ^2 }{ 2 (\sigma ^{(\pm )})^2 } \right] \end{aligned}$$

31 $$\begin{aligned} \left( \sigma ^{(\pm )} \right) ^2= & {} \left| \mathbf {k}\cdot \left( \delta \mathbf {U} \pm \delta \mathbf {V}_\mathrm{A} \right) \right| ^2 \end{aligned}$$

The random sweeping model distributes the fluctuation energy over the frequencies, and the fluctuation energy in the wavevector domain cannot be determined within the model. One may then postulate the inertial range spectrum for the Richardson–Kolmogorov scaling $E(k) \propto k^{-5/3}$ or the Iroshnikov–Kraichnan scaling $E(k) \propto k^{-3/2}$. The Kolmogorov scaling is used in Fig. 4.

The Alfvén waves may exist either forward or backward direction with respect to the mean flow or the mean magnetic field, or the counter-propagating waves may carry different fluctuation energies. The cross helicity $h_\mathrm{c}$ is the measure of the energy imbalance between the parallel and anti-parallel directions to the mean magnetic field:

32 $$\begin{aligned} h_\mathrm{c} = \langle \delta \mathbf {U} \cdot \delta \mathbf {V}_\mathrm{A} \rangle \,, \end{aligned}$$

where the angular bracket denotes the operation of the statistical averaging.

### Kinetic waves

**Fig. 5:**
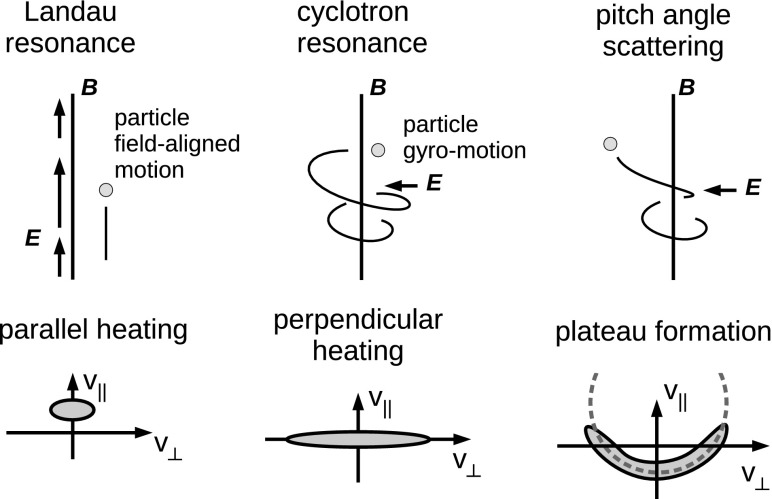
Sketches of wave–particle interactions in a plasma and the associated population in the velocity distribution function. Image reproduced with permission from Narita (2017b), copyright by the author

On smaller spatial scales at about ion or electron gyro-radii or inertial lengths, the linear mode waves become *kinetic*, exhibiting a dispersive and dissipative character due to various kinds of wave–particle interactions such as coherent scattering processes (like Landau or cyclotron resonance) or incoherent processes (like pitch-angle scattering). See sketches of wave–particle resonance and the associated population in the velocity distribution function in Fig. 5.

Dispersion means a broadening of wave packet in the direction of propagation. Dispersion is caused by the frequency-dependent (or wavenumber-dependent) group speed. Thus, waves are dispersive when the dispersion relation is curved in the wavenumber–frequency domain. An example is the parallel-propagating whistler mode (to the mean magnetic field). At very low frequencies (far below the ion gyro-frequency) the whistler mode behaves as an MHD fast mode and the dispersion relation is linear between the frequencies and the wavenumbers, $\omega \propto k$. The group speed $v_\mathrm {gr} = \partial \omega / \partial k$ is independent from the wavenumbers. At intermediate frequencies around the ion gyro-frequency and higher, the whistler frequencies depend on the wavenumbers quadratically, $\omega \propto k^2$. The group speed depends now on the wavenumbers, and becomes higher at larger wavenumbers.

The curved shape of the dispersion relation indicates that the ratio of the electric field to the magnetic field also depends on the wavenumbers, since the ratio is closely related to the phase speed, $v_\mathrm {ph} = \omega /k = \delta E/ \delta B$, due to the induction equation. The electric field amplitude becomes increasingly larger for a dispersive wave with an increasing sense of frequencies like the whistler mode. In a dispersive plasma, the fluctuation energy can be transferred from the magnetic field onto the electric field, or vice versa.

Dissipation means a temporal damping of fluctuating fields by binary collisions, finite resistivity, or wave–particles interactions. In a collisionless plasma, wave–particle interactions can occur in the longitudinal sense (Landau resonance) by accelerating particles that have a similar velocity with the wave phase speed (such that the interaction time becomes longer) as well as in the transverse sense by accelerating particles through a resonance in the cyclotron motion. Dissipation is essentially a heating process, and is considered as irreversible. Whistler waves at frequencies close the electron gyro-frequency can be in resonance with the electrons and are subject to the cyclotron damping.

The ion gyro-radius and the inertial length are of the order of several hundred to 1000 km under a typical condition of the solar wind at the Earth orbit (1 astronomical unit from the Sun), while that of the electrons are of the order of ten to hundred km. The picture of linear modes is useful in understanding fluctuations in space plasma. The ion-kinetic waves can be regarded as extensions of the MHD linear wave modes to smaller wavelengths, and can be grouped into the Alfvén mode, the fast mode, and the slow mode families.

**Fig. 6:**
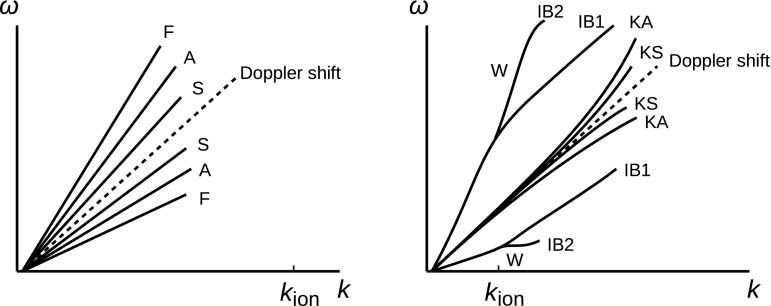
Sketches of dispersion relations in the Eulerian frame for magnetohydrodynamic waves (“F” for the fast mode, “A” for the Alfvén mode, and “S” for the slow mode) on the left panel and ion-kinetic waves obtained from the linear Vlasov theory for a quasi-perpendicular propagation to the mean magnetic field (“KS” for the kinetic slow mode, “KA” for the kinetic Alfvén mode, “W” for the whistler mode, “IB1” for the fundamental ion Bernstein mode, and “IB2” for the second harmonic of the ion Bernstein mode)

In the spirit of weak turbulence, linear mode waves are the primary constituent of turbulent fluctuations. Most of the fluctuation energy are stored along the dispersion relations in the wavenumber–frequency diagram as an “energy reservoir” Off-branch modes that have a deviation in frequency from the linear-mode dispersion relation are also possible. In the weak turbulence approach, the off-branch modes are considered to contain only a small amount of the fluctuation energy. The picture of Doppler-shifted dispersion relations as an “energy reservoir” can be extended from the Alfvén waves to the three magnetohydrodynamic wave modes (Aflvén, fast, and slow modes) with splitting into six branches (including both forward and backward propagation to the mean flow direction) or to the kinetic linear-mode waves with splittings, curves, and break-ups of the dispersion relations Fig. 6 displays the splitting of the energy spectrum peaks into the six magnetohydrodynamic wave modes at wavenumbers far below the ion inertial wavenumber $k_\mathrm{ion} = \varOmega _\mathrm{i}/V_\mathrm{A}$ (left panel) and the splitting into various branches of quasi-perpendicular propagating wave modes (to the mean magnetic field) in the ion-kinetic range at wavenumbers larger than the ion inertial wavenumber (right panel).

Each kinetic mode is obtained by a linearly perturbing the Vlasov equation (typically assuming a Maxwellian or a bi-Maxwellian plasma) and solving the equation for the wave electric field (which reduces to finding non-trivial roots for the wave dielectric tensor) under a given set of parameters like the wavenumber, the propagation angle to the mean magnetic field, the plasma parameter beta, the ion-to-electron temperature ratio, and the Alfvén speed with respect to the speed of light (Stix 1992; Gary 1993). The solution is obtained in the form of dispersion relation, that is, the frequency dependence in the complex number domain as a function of the wavenumbers or wavevectors. Analytic solutions are obtained only in few cases.

For a quasi-parallel propagation to the mean magnetic field, possible ion-kinetic modes (assuming a temperature isotropy) are the whistler mode, the ion–cyclotron mode, and the ion-acoustic mode. For a quasi-perpendicular propagation, there are four possible ion-kinetic modes as a transition of the quasi-parallel modes: the kinetic Alfvén mode, the kinetic slow mode, the oblique whistler mode, and the ion Bernstein mode. Those wave modes can be grouped into the Alfvén, fast, and slow mode families by tracking the dispersion relation onto the large-scale magnetohydrodynamic modes as follows.

#### Alfvén mode family

*Ion cyclotron mode*

Ion cyclotron mode is an extension of the Alfvén mode onto the ion kinetic scales. Polarization of the electric field is left-handed in the temporal sense and has the same rotation sense as the gyro-motion of ions when one views toward the mean magnetic field at a fixed position. In the cold plasma treatment, the ion cyclotron mode is referred to as the L mode for the left-hand circular polarization. The ion cyclotron mode has a resonance with the ion gyro-motion (and thus subject to damping). The frequency approaches the resonance frequency asymptotically from the side of lower frequencies in the dispersion relation. The resonance frequency is the ion cyclotron frequency for for the parallel or anti-parallel propagation (to the mean magnetic field). For oblique propagation directions, the resonance frequencies become lower at larger propagation angles from the mean magnetic field. The dispersion relation of the ion cyclotron mode in the cold plasma treatment is (Stix 1992):

33 $$\begin{aligned} \frac{\omega }{\varOmega _\mathrm{i}} = \left[ 1 + \frac{\varOmega _\mathrm{i}^2}{k_\Vert ^2 V_\mathrm{A}^2} + \frac{\varOmega _\mathrm{i}^2}{k^2 V_\mathrm{A}^2} \right] ^{-1} \end{aligned}$$

The resonance frequency also becomes lower a larger value of beta. The ion–cyclotron mode can propagate along the magnetic field line and thus transmit the energy over a large distance along the field line. The existence of the ion cyclotron mode is reported in various regions in space plasma, for example, waves falling from the radiation belt down to the Earth ionosphere causing pulsating proton aurora (Nomura et al. 2012). Ions are not only accelerated by the cyclotron resonance but are also pitch-angle scattered by the ion cyclotron mode. Solar wind protons show arc structures in the velocity distribution function, centered at the apparent phase speed of the ion cyclotron mode, which indicates the existence of the ion cyclotron mode in the inner heliosphere (Marsch and Tu 2001). Also, numerical simulations show that the ion cyclotron mode can interact with the helium alpha particles Maneva et al. (2013, 2015); Ofman et al. (2014); Ozak et al. (2015).

*Kinetic Alfvén mode*

Kinetic Alfvén mode is a quasi-perpendicular limit of the ion–cyclotron mode in the linear Vlasov theory. At propagation angles of about 70–80$^\circ$ from the mean magnetic field, the ion cyclotron mode has a transition in the electric field polarization and the curvature sense of the dispersion relation (or the rate of frequency change at increasingly larger values of wavenumbers) becomes from a converging or a resonant sense (convex sense of the dispersion branch) into a diverging sense of the frequency increase (concave sense of the dispersion branch). The kinetic Alfvén mode has very low frequencies and weakly damped at around the ion inertial length. At sufficiently small wavelengths than the ion inertial length, the kinetic Alfvén mode is again subject to damping. The dispersion relation of the kinetic Alfvén mode is analytically obtained from the two-fluid model of plasma (Lysak and Lotko 1996; Hollweg 1999; Lysak 2008):

34 $$\begin{aligned} \frac{\omega }{\varOmega _\mathrm{i}}= & {} \frac{k_\Vert V_\mathrm{A}}{\varOmega _\mathrm{i}} \left( 1 + k_\perp ^2 r_\mathrm{gi}^2 \right) ^{1/2} \end{aligned}$$

35 $$\begin{aligned}= & {} \frac{k_\Vert V_\mathrm{A}}{\varOmega _\mathrm{i}} \left[ 1 + \frac{\beta }{2} \left( \frac{k_\perp V_\mathrm{A}}{\varOmega _\mathrm{i}} \right) ^2 \right] ^{1/2} \,, \end{aligned}$$

where $r_\mathrm{gi} = v_\mathrm{th(i)}/\varOmega _\mathrm{i}$ is the gyro-radius for the thermal ions and the plasma beta is related to the ion thermal speed $v_\mathrm{th{i}}$ through the relation $\beta /2 = (v_\mathrm{th}/V_\mathrm{A})^2$. The kinetic Alfvén mode is considered as one of the most relevant wave modes in ion-kinetic scale turbulence such as in the solar wind, since the frequencies are so low (by far smaller than the ion cyclotron frequency) and the waves cannot decay onto even lower frequencies by wave–wave couplings. Also, the kinetic Alfvén mode is only moderately damped at the ion inertial length. Various in situ observations of turbulent fluctuations in the solar wind are favorably interpreted as the kinetic Alfvén mode from 0.1 to 100 Hz in the spacecraft frame (Bale et al. 2005; Sahraoui et al. 2010; Salem et al. 2012; TenBarge et al. 2012; Chen et al. 2013; Kiyani et al. 2013; Roberts et al. 2013). On the other hand, the dominance of the kinetic Alfvén mode is subject to the plasma parameter beta. Recent numerical simulations for forced hybrid kinetic turbulence suggest that the kinetic Alfvén mode dominates ion-scale turbulence in a high-beta plasma, while the whistler mode dominates in a low-beta plasma Cerri et al. (2016).

#### Fast mode family

*Whistler mode*

Whistler mode is smoothly connected from the fast mode with a right-hand temporal rotation sense around the mean magnetic field (Gary 1986). In the cold plasma treatment, the whistler mode is referred to as the R mode for the right-hand polarization. The existence of the whistler mode has been reported in various regions in space plasmas. The reason for this is that the whistler mode can exist in a wide range of frequencies (up to the electron cyclotron frequency) and propagation angles. Examples are magnetospheric chorus waves (high-frequency whistler) (Katoh 2014), magnetotail right-hand waves (Tsurutani and Smith 1984; Tsurutani et al. 1985), magnetosheath or magnetospheric lion roar waves (Baumjohann et al. 1999, 2000), magnetopause whistler waves (Vaivads et al. 2007), and waves departing from magnetic reconnection (Eastwood et al. 2009). In the ion-kinetic domain, the frequencies of the whistler mode are increasingly higher at larger wavenumbers, and the dispersion relation is analytically expressed as (Gary 1993):

36 $$\begin{aligned} \frac{\omega }{\varOmega _\mathrm{i}} = \frac{k V_\mathrm{A}}{\varOmega _\mathrm{i}} \left( 1 + \frac{ k_\Vert ^2 V_\mathrm{A}^2 }{ \varOmega _\mathrm{i}^2 } \right) ^{1/2} . \end{aligned}$$

The whistler mode becomes resonant with the electron gyro-motion at the resonance frequency. For the parallel (or anti-parallel) propagation, the resonance frequency is the electron cyclotron frequency. For oblique propagations, the resonance frequency becomes lower down to the lower hybrid frequency. There is an increasing amount of evidence for the existence of the whistler mode on the spatial scales between ion-kinetic and electron-kinetic ones (Narita et al. 2016; Stansby et al. 2016). The whistler mode is more difficult to find in solar wind turbulence. The reason for this lies in that the waves at lower frequencies tend to attain larger amplitudes such that the kinetic Alfvén mode is first detected when analyzing large-amplitude (and incompressible) components.

*Ion Bernstein mode*

Ion Bernstein mode appears when the dispersion branch of the whistler mode begins to break up at resonance at the ion cyclotron frequency and its harmonics:

37 $$\begin{aligned} \frac{\omega }{\varOmega _\mathrm{i}} \simeq n (n=1, 2, \cdots ) . \end{aligned}$$

On the other hand, the frequencies between different ion Bernstein modes still retain the dispersion branch for the whistler mode. In a low-beta plasma, the quasi-perpendicular whistler mode is more smoothly connected from lower frequencies to higher frequencies. In a high-beta plasma, in contrast, the whistler mode branch almost vanishes because of the resonant splitting into the ion Bernstein mode. The Bernstein mode has both electrostatic and electromagnetic components. and shows a variety of wave–wave coupling realizations due to many branches at different frequencies (Jenkins et al. 2013). The ion Bernstein mode is a subject of extensive studies in Tokamak, fusion, and laboratory plasmas (Cardinali et al. 2002; Intrator et al. 2003; Toida et al. 2004; Korsholm et al. 2011; Zhang et al. 2012). In the solar wind, there are indications that some wave components agree with the dispersion relations of the ion Bernstein mode (Perschke et al. 2013, 2014).

*Lower hybrid mode*

The lower hybrid mode is obtained as a resonance frequency of the whistler mode in the limit of perpendicular propagation to the mean magnetic field. It is unique in the lower hybrid mode that the wave can heat or accelerate both electrons by the cyclotron resonance and ions by the perpendicular Landau resonance. The dispersion relation of the lower hybrid mode involves all the fundamental frequencies (cyclotron frequencies for electrons and ions, $\varOmega _\mathrm{e}$ and $\varOmega _\mathrm{i}$, respectively; and plasma frequencies for electrons and ions, $\omega _\mathrm{pe}$ and $\omega _\mathrm{pi}$, respectively). The analytic expressions in the cold plasma treatment is (Marsch and Chang 1982, 1983):

38 $$\begin{aligned} \frac{\omega }{\varOmega _\mathrm{i}} = \frac{\omega _\mathrm{pi}}{\varOmega _\mathrm{i}} \left[ \frac{ 1 + \left( \frac{ k_\Vert \omega _\mathrm{pe} }{ k \omega _\mathrm{pi} } \right) ^2 }{ 1 + \left( \frac{ \omega _\mathrm{pe} }{ \varOmega _\mathrm{e} } \right) ^2 } \right] ^{1/2}. \end{aligned}$$

Note that the frequency ratio $\omega _\mathrm{pi}/\varOmega _\mathrm{i}$ is equivalent to the ratio of the speed of light to the Alfvén speed, $c/V_\mathrm{A}$. In the solar wind, $\omega _\mathrm{e}^2 \gg \varOmega _\mathrm{e}^2$ holds and the dispersion relation [Eq. (38)] reduces to a single-frequency, system oscillation mode (i.e., no dependence on the wavenumbers) at the lower hybrid frequency, $\omega \simeq |\varOmega _\mathrm{e} \varOmega _\mathrm{i}|^{1/2}$, or the mass ratio of ions to electrons in the normalized units,

39 $$\begin{aligned} \frac{\omega }{\varOmega _\mathrm{i}} \simeq \left| \frac{ \varOmega _\mathrm{e} }{ \varOmega _\mathrm{i} } \right| ^{1/2} = \sqrt{\frac{m_\mathrm{i}}{m_\mathrm{e}}} . \end{aligned}$$

The lower hybrid mode is observed in a thin current layer at the Earth magnetopause (Vaivads et al. 2004) and on the magnetic reconnection site (ion diffusion region) (Graham et al. 2017). Also, the lower hybrid mode can contribute to the anomalous resistivity (resistivity in a collisionless medium) through the wave–particle interactions and the current filament formation (Bingham et al. 2004).

#### Slow mode family

*Ion acoustic mode*

Ion acoustic mode is a electrostatic mode, but can exist in a magnetized plasma if the propagation is aligned with the mean magnetic field direction. The oscillation is longitudinal. The dispersion relation is linear:

40 $$\begin{aligned} \omega = k_\Vert c_\mathrm{s} \,, \end{aligned}$$

where the sound speed is defined in the kinetic fashion as (Gary 1993)

41 $$\begin{aligned} c_\mathrm{s} = \sqrt{ \frac{ T_\mathrm{e} + 3 T_\mathrm{i} }{ m_i } } \end{aligned}$$

*Kinetic slow mode*

Kinetic slow mode is a small-wavelength extension of the slow mode to the ion kinetic range, and has as low frequencies as that of the kinetic Alfvén mode (depending on the value of beta). While the slow mode is generally considered as a strongly Landau-damped mode, the kinetic slow mode has quasi-perpendicular wavevectors to the mean magnetic field and is only moderately damped at around the ion inertial length. The dispersion relation for the kinetic slow mode is (Zhao et al. 2014):

42 $$\begin{aligned} \frac{\omega }{\varOmega _\mathrm{i}}= & {} \frac{k_|}{k_\perp } \left( \frac{ k_\perp ^2 r_\mathrm{gi}^2 }{ 1 + k_\perp ^2 r_\mathrm{gi}^2 } \right) ^{1/2} \end{aligned}$$

43 $$\begin{aligned}= & {} \frac{k_\Vert }{k_\perp } \left( \frac{\beta }{2} \right) ^{1/2} \left[ 1 + \frac{2}{\beta } \left( \frac{ k_\perp V_\mathrm{A} }{ \varOmega _\mathrm{i} } \right) ^{-2} \right] ^{-1} \end{aligned}$$

Compressive fluctuations in the solar wind may be slow mode waves (Howes et al. 2012; Klein et al. 2012) or pressure-balanced structures between the magnetic field and the plasma in the solar wind (Yao et al. 2013; Zhao et al. 2014) may be a realization of the kinetic slow mode.

### Zero-frequency mode

The zero-frequency mode represents a non-propagating perturbation of density and temperature (Kadomtsev 1960). The entropy mode does not change the total plasma pressure nor the specific entropy on the perturbation Since the frequencies are zero at various wavenumbers, the propagation speeds (phase speed) are also zero in the plasma rest frame (co-moving with the mean flow).

### Sideband waves

The energy spectrum in the wavenumber–frequency domain shows the sideband wave activity whenever the condition for the ideally convection (Doppler shift) or that for the plane waves breaks down. The sideband waves have a frequency deviation from the linear mode dispersion relation, and the energy spectrum shows a spread around the spectral peak. The Doppler broadening is one example, but there are a variety of reasons and mechanisms for the excitation of the sideband waves: the random sweeping by the large-scale flow variations or the large-scale waves (both cause the Doppler broadening), the excitation of sideband waves by wave–wave interactions, the wave damping effect, and the wave packet formation.

In the random sweeping case, the large-scale fluctuations in the flow velocity and the magnetic field are assumed to be Gaussian in the time domain, and the frequency slice of the energy spectrum at a given wavenumber also shows a Gaussian frequency distribution

44 $$\begin{aligned} E(k,\omega ) = \frac{E(k)}{\sqrt{2\pi \sigma ^2}} \exp \left[ -\frac{ (\omega - \omega _0)^2 }{ 2\sigma ^2 } \right] \end{aligned}$$

The random sweeping model assumes that the small-scale turbulent fluctuations are advected by the large-scale flow velocity without affecting the large-scale field. If the large-scale field is also in a turbulent state, the frequency spread shows a non-Gaussian shape such as a kappa distribution for intermittent fluctuations.

**Fig. 7:**
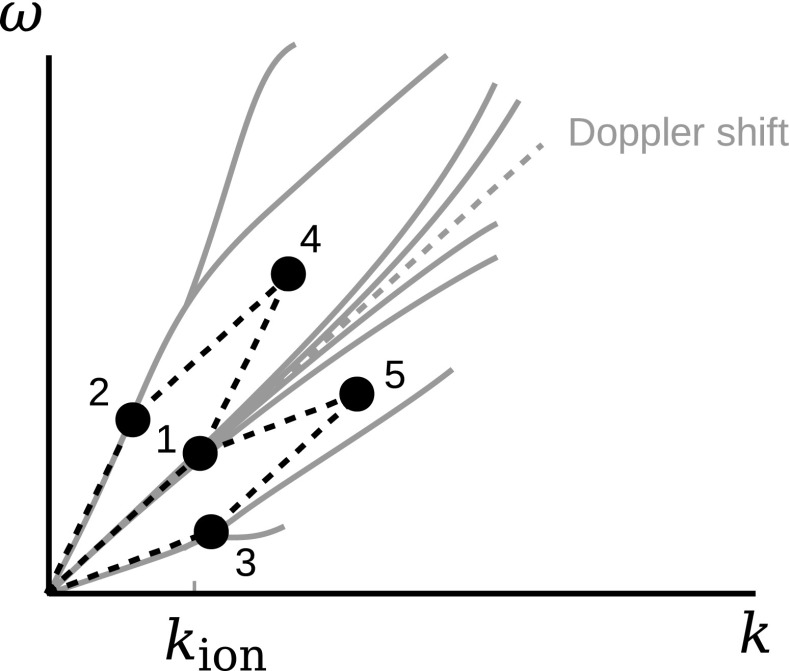
Three-wave couplings in the dispersion relation diagram (parallelograms in the wavenumber–frequency domain) superposed onto the ion-kinetic wave modes. The initial waves (1, 2, and 3 in the digram) interact and generate the wave 4 and the wave 5 through the coupling of the waves 1 and 2, and that of the waves 1 and 3

The sideband waves can be excited through wave–wave interactions. One may introduce a three-wave coupling as the simplest treatment under the condition of the frequency resonance and the wavevector resonance:

45 $$\begin{aligned} \omega _\mathrm{a} + \omega _\mathrm{b}= & {} \omega _\mathrm{c} \end{aligned}$$

46 $$\begin{aligned} \mathbf {k}_\mathrm{a} + \mathbf {k}_\mathrm{b}= & {} \mathbf {k}_\mathrm{c} \,, \end{aligned}$$

where the indices “a” and “b” represent the two interacting waves, and the index “c” is the generated wave. An example of sideband wave formation through the wave–wave interactions is the modulational instability (Mio et al. 1976; Mjølhus 1976; Nariyuki and Hada 2006) in which all the participating waves propagate in the same direction (e.g., parallel to the mean magnetic field) and one participating wave has a sufficiently low frequency or a long wavelength such that the generated wave has a frequency or a wavenumber close to that of the other participating wave. The sideband formation by the three-wave couplings are displayed in Fig. 7. The wave component 1, 2, and 3 are present at the initial time. The wave component 1 nearly Doppler-shifted structure (or a zero-frequency mode) and the component 2 is on a linear mode branch. The coupling of the wave component 1 with the component 2 generates a sideband wave (the component 3) and the coupling of the component 1 with that of 3 generates another sideband wave, the component 5.

The wave form in the time domain can also appear as sideband waves in the spectral domain. An example is a Gaussian wave packet. The Fourier transform of a Gaussian distribution (e.g., in the time domain) is again Gaussian (in the frequency domain), so both the random sweeping and the Gaussian wave form result in a Gaussian shape of the frequency-sliced spectrum (at a given value of the wavenumber). Another scenario is wave damping. The Fourier transform of a temporally decaying wave results in a Lorentzian or Breit–Wigner distribution in the frequency domain, so the spectrum is expressed as:

47 $$\begin{aligned} E(k,\omega ) = \frac{E(k)}{(\omega - \omega _0)^2 + \gamma ^2} \,, \end{aligned}$$

where $\omega _0$ is again the linear mode frequency, and $\gamma$ the damping rate. If the Breit–Wigner distribution is assumed on the data analysis, it is possible to estimate the damping rate from the measurement of the frequency-slice of the spectra.

### Coherent structures

Turbulent fluctuations are not fully random but must necessarily have both random phases and coherent phases. For example, the resonance conditions for the wave–wave couplings [Eqs. (45)–(46)] a coherent process because the phase of the generated wave is automatically determined by that of the initial waves, including the initial phase. Coherence in the phase results in a structure formation. Coherent structures appear as non-propagating, standing structures in the turbulent fields, and are merely swept by the mean flow.

There are a variety of coherent structure types. Eddies are the fundamental constituent of hydrodynamic turbulence and originate in the advection term of the Navier–Stokes equation. If the medium is compressible, shock waves or density cavity can occur. In the case of plasma turbulence, one-dimensional current sheet can be formed with various thicknesses down to the electron gyro-radius. If the current sheet is sufficiently thin, magnetic reconnection sets on and generates bursty flows. The electric current and the magnetic field can confine the plasma and form flux tubes, flux ropes, and force-free type spiral magnetic field structures. In the shock-downstream region such as the magnetosheath region of planetary magnetospheres, the mirror instability sets on due to the over-heated plasma in the perpendicular direction to the mean magnetic field and the pressure balanced structure is formed, making a balance between the thermal pressure and the magnetic pressure.

**Fig. 8:**
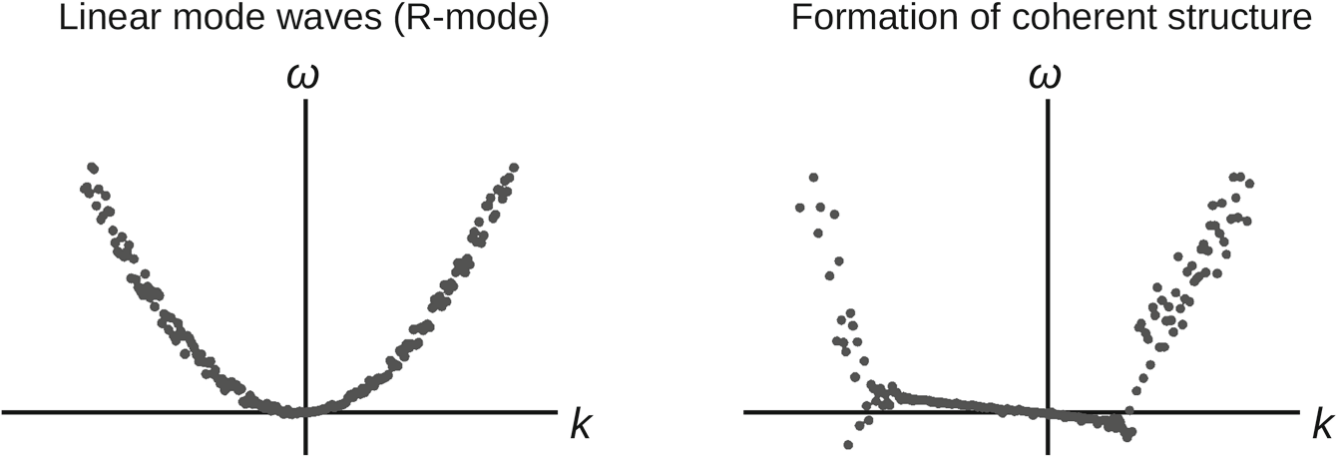
Transition from linear mode waves into a formation of coherent structure by re-organizing the dispersion relation with a constant group speed over a wide range of wavenumbers. Image adapted from Nariyuki and Hada (2006)

An important feature of the coherent structure is the phase coherence or phase synchronization. An example is a Dirac delta function which can be decomposed into a set of plane waves by the Fourier transform. All the constituent waves have the same amplitude (which is unity) and the same phase. An easy check of the phase coherent is to browse the waveform in the time domain or the spatial domain. If the coherent structure is a Gaussian bell, the Fourier transform of the wave field is again a Gaussian. So, bell-shaped coherent structures have a certain bandwidth or a range of wavenumbers in which the wave phases are synchronized. If the structure moves without changing its form, the group velocity of the waves must be the same. Such a propagating coherent structure no longer follows the dispersion relation for the linear mode. An example of phase coherent seen in the dispersion relation diagram is shown in Fig. 8. The system (Hall-MHD system) develops from the linear-mode waves into a formation of coherent structure with instantaneous or short-living synchronization in the dispersion relation by violating the linear mode (Nariyuki and Hada 2006). Since the coherent structure retains its waveform with a finite propagation speed (group speed), the coherent structure is represented by a linear relation between the frequencies and the wavenumbers.

### Lessons from the observations

#### Eulerian picture

**Fig. 9:**
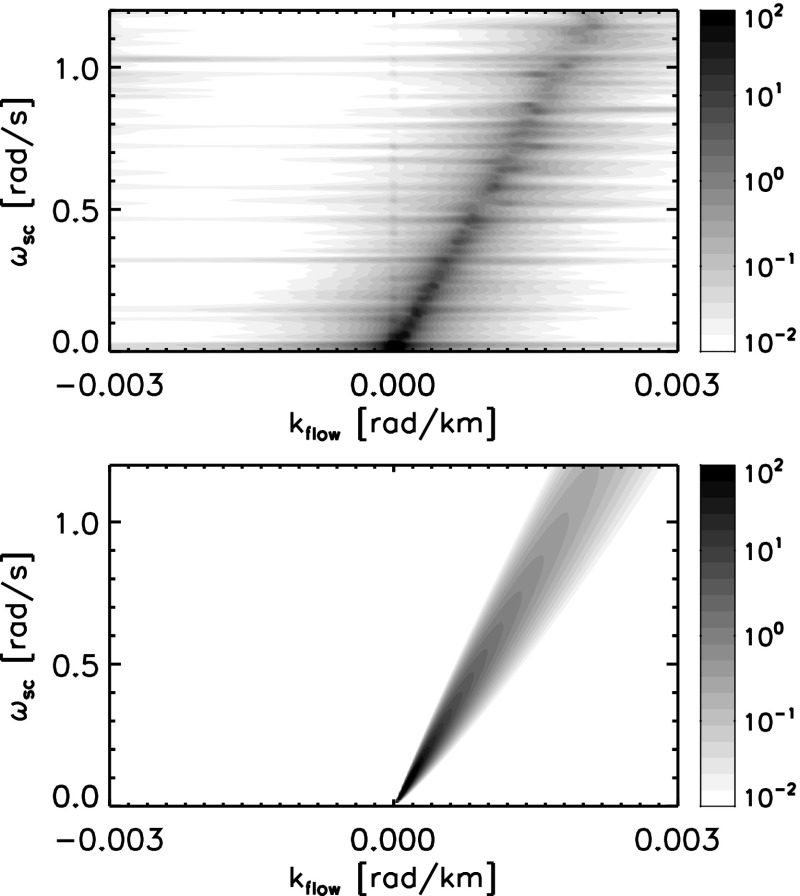
Energy spectrum for magnetic field fluctuations in the streamwise wavenumber–frequency domain obtained from the Cluster spacecraft data in the solar wind (top) and reconstruction after a fitting in the mean flow velocity and the root-mean-square flow velocity. Image reproduced with permission from Narita (2014), copyright by the author

The energy spectrum in the wavenumber–frequency domain (in the streamwise sense) can be determined by multi-spacecraft measurements in the solar wind (Fig. 9 top panel). Reconstruction of the spectrum after a fitting of the random sweeping model against the measured spectrum is displayed in the bottom panel. While most of the fluctuation energy is stored along the Doppler shift with the relation $\omega = k_\mathrm {flow} U_0$, the sense of the frequency broadening is different. The measured spectrum exhibits nearly constant frequency broadening around the Doppler shift at various wavenumbers. The random sweeping model predicts that the frequency broadening becomes larger at higher wavenumbers. The difference in the frequency broadening sense may come from the observational effects (limited band-width in the wavenumber domain due to a limited spatial sampling points) or from the breakdown of the Gaussian statistics assumption on the larger scales.

#### Catalogue of dispersion diagrams

Dispersion relation diagrams can be measured in situ in space plasmas either using multiple spacecraft data or using both the electric field and the magnetic field.

- Using multi-spacecraft data, the dispersion relation diagrams are obtained by determining frequencies from the time series data (with the help of Fourier transform) and wavevectors from the phase difference from an sensor to another. There are various ways to efficiently determine the wavevectors from multi-spacecraft data, e.g. , the timing or phase differencing method (Hoppe and Russell 1983; Dudok de Wit et al. 1995), the minimum variance projection such as the wave telescope or k-filtering projection (Capon 1969; Neubauer 1990; Pinçon and Lefeuvre 1991; Motschmann et al. 1996; Glassmeier et al. 2001), and the eigenvalue-based projection (Schmidt 1986). Also, the correction for the Doppler shift $\mathbf {k} \cdot \mathbf {U}$ is possible once the wavevector $\mathbf {k}$ and the bulk flow velocity $\mathbf {U}$ are known to interpret the frequencies in the rest frame of plasma, co-moving with the bulk flow.
- Using the electric and magnetic field data, one computes the ratio of the electric field to the magnetic field. This ratio is a phase speed (in the observer frame) when Fourier transforming the induction equation, $v_\mathrm {ph} = \omega /k = \delta E_\mathrm {tr1} / \delta B_\mathrm {tr2}$. See, e.g., Bale et al. (2005) or Eastwood et al. (2009) for applications. It is important to note here that two mutually-orthogonal transverse components to the wave propagation direction (wavevector direction) need to be used to estimate the phase speed and that one assumes only one wave mode at a given frequency (i.e., only one dispersion branch). Otherwise the estimate of the phase speed may be mixed up with the electrostatic component or be influenced by multiple dispersion branches.

**Fig. 10:**
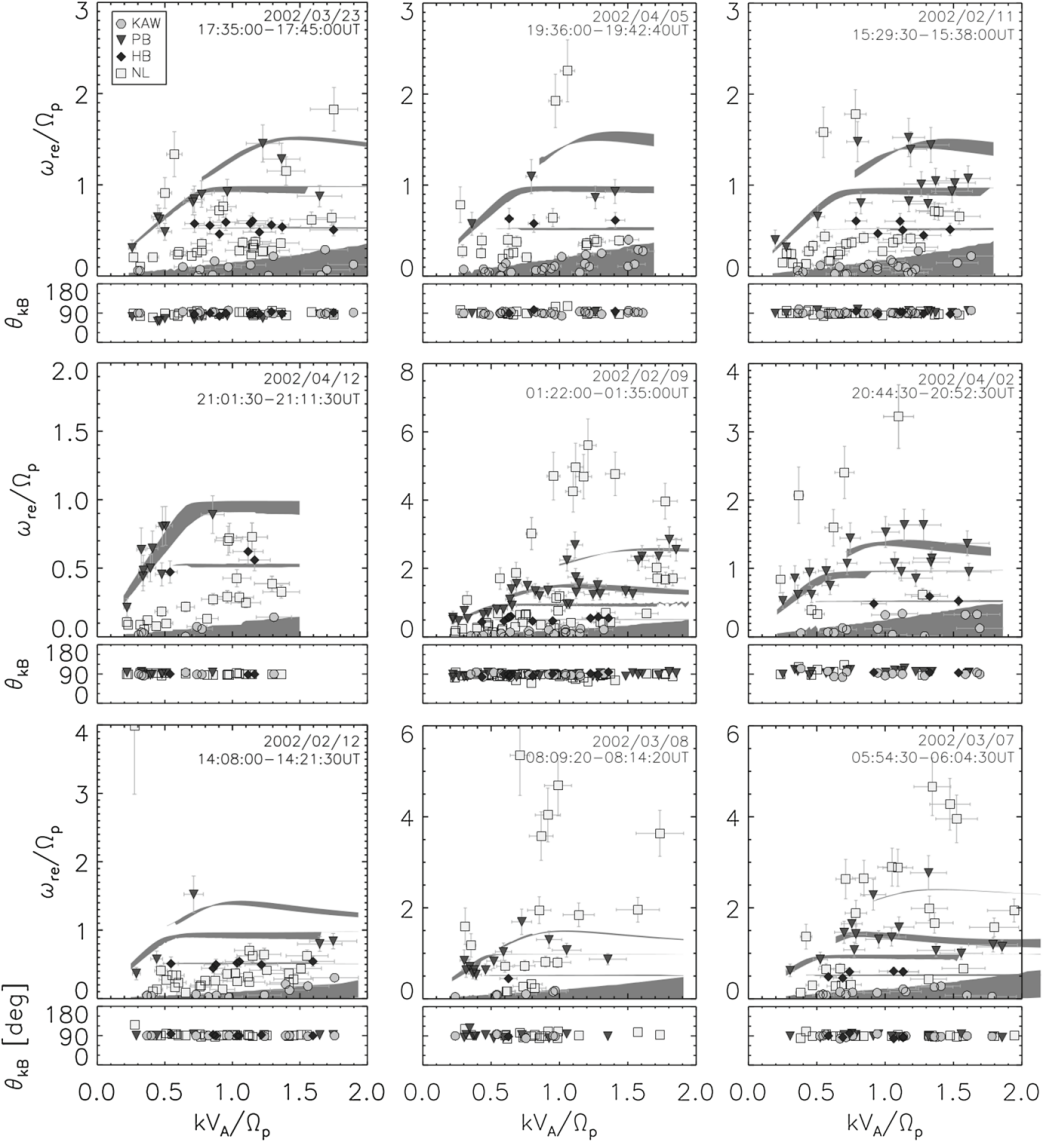
Dispersion relation diagrams and propagation angles observed in the solar wind. The detected wave components (a set of the frequencies and the wavenumbers) are associated with different wave modes: kinetic Alfvén mode (in circles), ion Bernstein mode for protons (in triangles) and for helium alpha particles (in diamonts), and non-linear or sideband mode (in squares). Image reproduced with permission from Perschke et al. (2014), copyright by AAS

Figure 10 displays nine samples of dispersion relation diagram in the solar wind on spatial scales around ion kinetic motion (around ion inertial length, typically at about 400 km). The diagrams are obtained by the following procedure. Four-point magnetic field data from the Cluster spacecraft are used here. The magnetic field data are Fourier-transformed from the time domain into the spacecraft-frame frequency domain for each spacecraft, and are further projected from the spatial domain onto the wavevector domain to obtain the $3\times 3$ spectral density matrix as a function of spacecraft frequencies and wavevectors. The projection of the fluctuating fields onto the wavevector domain is achieved by a combination of the minimum variance projection with the eigenvalue-based decomposition the multi-point signal resonator technique (Narita et al. 2011). The local peaks of the energy spectra in the three-dimensional wavevector domain are identified at each frequency (in the spacecraft-frame of reference) by scanning the total fluctuation energy (trace of the spectral density matrix) and a set of the frequencies and the wavevectors are obtained. Fluctuating fields are assumed to be composed of a set of plane waves and noise without assuming or imposing any dispersion relation in the data. The frequencies are transformed from the spacecraft frame into the plasma rest frame co-moving with the mean bulk flow speed by correcting for the Doppler shift. The rest-frame frequencies are normalized to the ion cyclotron frequency as $\omega _\mathrm {re}/\varOmega _\mathrm {i}$, and the wavenumbers (magnitude of the wavevector) to the ion inertial wavenumber as $k V_\mathrm {A}/\varOmega _\mathrm {i}$. Finally, the dispersion relations for theoretically predicted linear modes are over-plotted using the values of propagation angle $\theta _\mathrm {kB}$ to the mean magnetic field (averaged over the wavevector domain) and ion beta from the measurements. Propagation directions are highly oblique (almost perpendicular) to the mean magnetic field in the solar wind. The detected wave components (a set of the frequencies and the wavenumbers) are associated with different wave modes: kinetic Alfvén mode (in circles), ion Bernstein mode for protons (in triangles) and for helium alpha particles (in diamonts), and non-linear or sideband mode (in squares). Dispersion relations including a variation or an uncertainty of propagation angles are also displayed for kinetic Alfvén mode (the lowest frequencies), helium-alpha Bernstein mode (the second lowest at a half of the proton cyclotron frequency), proton Bernstein mode at the proton cyclotron frequency and the second harmonic.

The detected waves are associated to different linear modes including uncertainties in the Doppler shift correction. The detected waves that have large deviations in frequency from the linear mode ones are grouped into the sideband waves or nonlinear waves. While about 25% of the wave population is associated with the kinetic Alfvén mode and another 25% with the ion Bernstein mode for protons, about 40% of the wave population are outside the frequency ranges expected from the linear Vlasov theory and represent the sideband waves. Dispersion analysis shows various examples of linear mode waves: kinetic Alfvén mode (Sahraoui et al. 2010; Roberts et al. 2015a), ion Bernstein modes (Perschke et al. 2013), whistler mode (Narita et al. 2011). Whether those “off-branch” waves are instantaneous waves generated by wave–wave interactions (which are presumably damped quickly) or a fragment of propagating coherent structure remain unsolved. A test for phase coherence or a study of waveform will be helpful to understand the physical process of the sideband or off-branch waves in the observations.

#### Statistical dispersion diagram

**Fig. 11:**
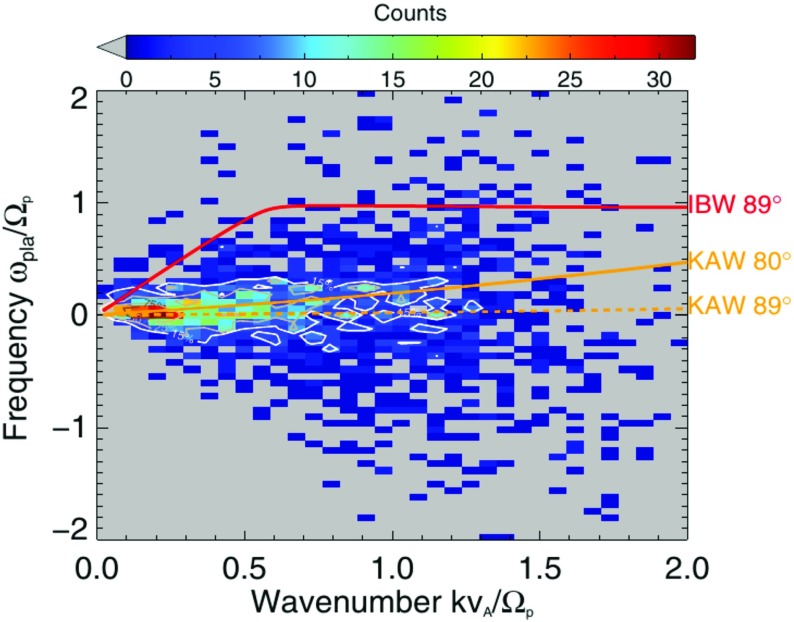
Histogram of the discrete wave components (local peaks in the energy spectra) in the domain spanning the wavenumbers and the rest-frame frequencies. The wavenumbers are normalized to the ion inertial length for protons as $k V_\mathrm {A}/\varOmega _\mathrm {p}$ and the frequencies to the ion cyclotron frequency as $\omega /\varOmega _\mathrm {p}$. Dispersion relations for the kinetic Alfvén mode (KAW) and the ion Bernstein mode (for protons) are over-plotted. Image reproduced with permission from Roberts et al. (2015b), copyright by AAS

Dispersion analysis can be applied to various solar wind events to construct the dispersion relation diagram statistically. Figure 11 displays a histogram of the discrete wave components using 52 intervals of solar wind in the Cluster spacecraft data (Roberts et al. 2015b). The spread in the rest-frame frequencies is not small, extending to the ion cyclotron frequency in the both signs. Here, a positive frequency in the rest frame indicates the anti-sunward propagation and a negative frequency the sunward propagation. Wavevector directions are highly oblique, and two modes are compared on the diagram: the kinetic Alfvén mode and the ion Bernstein mode (fundamental mode for protons). The peak of the distribution of the discrete wave components has nearly zero frequencies and falls onto with the dispersion relation branch of the kinetic Alfvén mode. However, many discrete wave components have significant deviation from the linear mode branches or frequencies. Turbulent fluctuations in the solar wind are thus composed both of the linear mode waves and the sideband or nonlinear mode fluctuations.

An observational model of the energy spectrum also constructed in the wavenumber–frequency domain using 31 intervals of solar wind in the Cluster spacecraft data (Perschke et al. 2016) [a different set of intervals from Roberts et al. (2015b)]. The spectrum model assumes the Gaussian frequency broadening as follows.

48 $$\begin{aligned} E(\tilde{k}_\perp ,\tilde{\omega })= & {} E(\tilde{k}_\perp ) F(\tilde{k}_\perp ,\tilde{\omega }) \end{aligned}$$

49 $$\begin{aligned} E(\tilde{k}_\perp )= & {} \tilde{k}_\perp ^{-\alpha } \end{aligned}$$

50 $$\begin{aligned} F(k_\perp ,\omega )= & {} \frac{1}{\sqrt{2\pi \tilde{\sigma }^2(k)}} \exp \left[ -\frac{ \left( \tilde{\omega } - \tilde{\omega }_0(k) \right) ^2 }{ 2 \tilde{\sigma }^2(k) } \right] \end{aligned}$$

Here, the wavevector is in the perpendicular direction to the mean magnetic field, and *C* is a proportionality constant for the one-dimensional spectrum. The wavenumbers and the frequencies (in the plasma rest frame) are normalized to the ion scale as $\tilde{k} = k V_\mathrm {A}/\varOmega _\mathrm {i}$ and $\tilde{\omega } = \omega /\varOmega _\mathrm {i}$. The free parameters in the model are the spectral index $\alpha$, the frequency center $\tilde{\omega }_0$, and the frequency broadening $\tilde{\sigma }$. The spectral index is determined by the fitting procedure in two different wavenumber domains, $\alpha _\mathrm {i}$ for the MHD inertial range spectrum in the solar wind (typically for $\tilde{k} < 1$) and $\alpha _\mathrm {d}$ for the dispersive–dissipative range spectrum on the ion-kinetic scales (typically $\tilde{k} \ge 1$):

51 $$\begin{aligned} \alpha _\mathrm {i}= & {} 1.75 \pm 0.16 \end{aligned}$$

52 $$\begin{aligned} \alpha _\mathrm {d}= & {} 2.62 \pm 0.41. \end{aligned}$$

The frequency center is obtained from the data, and it is sufficiently close to zero:

53 $$\begin{aligned} \tilde{\omega }_0 = 0 . \end{aligned}$$

The frequency broadening (normalized to the ion cyclotron frequency, $\tilde{\sigma } = \sigma /\varOmega _\mathrm {i}$) is determined using a power-law to the wavenumber:

54 $$\begin{aligned} \tilde{\sigma } = \tilde{k}_\perp ^{1.67\pm 0.20} . \end{aligned}$$

## Wavevector anisotropy

### Impact of the large-scale magnetic field

Plasma turbulence is intrinsically anisotropic whenever the large-scale magnetic field is present. On the individual particle level, the Lorentz force ($q \mathbf {v}\times \mathbf {B}$, where *q* is the electric charge, $\mathbf {v}$ the particle velocity, and $\mathbf {B}$ the magnetic field) acts on the charged particle in the perpendicular components of the velocity to the magnetic field. On the fluid scale, the Lorentz force ($\mathbf {j} \times \mathbf {B}$) plays an important role in the plasma dynamics, contributing as the magnetic pressure gradient force and the magnetic tension. The anisotropic nature in the plasma dynamics also influences the energy transfer process in turbulence from one scale to another as well as the structure formation in the turbulent field.

Homogeneous and isotropic fluid turbulence is, in contrast, essentially composed of eddies with the vorticity axes in various directions and in various magnitudes. The turbulence energy transport is carried by the interactions between eddies generating eddies again with the vorticity axes in various directions and in various magnitudes. The interaction of eddies does not recognize the large-scale structure (except for a situation near the boundary or the wall of turbulent flow) in the inertial range.

Plasma turbulence has a larger degree of freedom (or control parameters) in that not only eddies but also electromagnetic waves such as Alfvén waves can interact with one another, and the wave–wave interactions are an additional energy carrier for the turbulent cascade. Electromagnetic waves in the plasma are so diverse. The wave mode or the dispersion relation is a function of the plasma parameter beta and the propagation angle. Furthermore, the fluctuation sense is also diverse such as the right-handed or left-handed rotation sense of the wave field and the compressible or incompressible sense of fluctuation with respect to the magnetic field direction.

Anisotropy may enter plasma turbulence in various ways, e.g, in the energy spectra, in the fluctuating sense, in the energy transfer rate, and in the dissipation rate. We limit ourselves this section to the anisotropic structure formation in turbulence. Anisotropy appears as extended or elongated structures of the energy spectrum in the wavevector domain spanning the parallel and the perpendicular components to the mean magnetic field (assuming, for simplicity, that the mean field can nearly be regarded as constant). Even with single spacecraft measurements, there are indications that the energy spectrum be anisotropic with respect to the mean magnetic field, for example, the change in the spectral index as a function of the projection angle (or the flow direction) to the mean magnetic field in Fig. 2 (Horbury et al. 2008; Osman and Horbury 2009) or the change in the correlation length with respect to the mean field (Matthaeus et al. 1990; Dasso et al. 2005).

### Two-component model

The presence of the large-scale or mean magnetic field causes two particular effects in plasma dynamics, which leads to a picture of two competing fluctuation geometries in the wavevector domain.

**Fig. 12:**
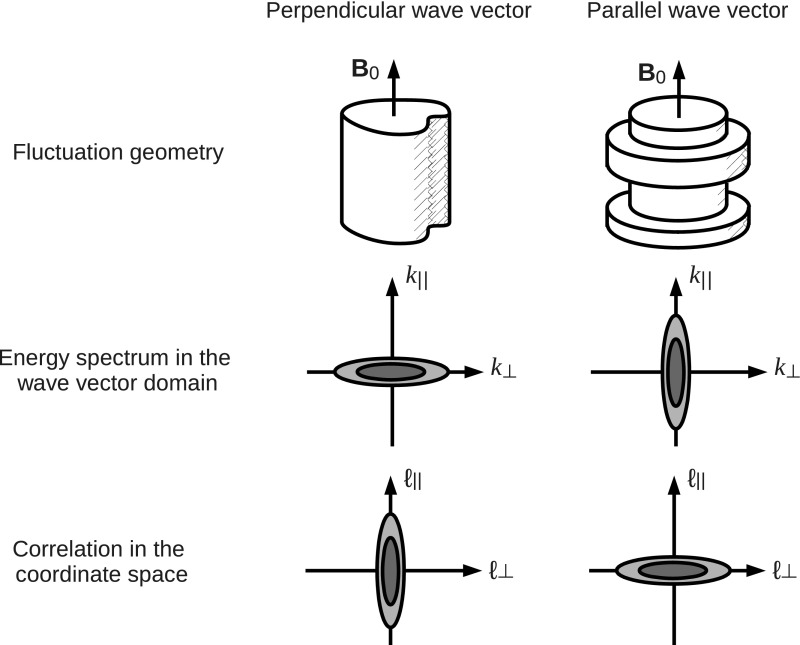
Fluctuation visualizations, the energy spectra in the wavevector domain, and the spatial correlations for the perpendicular wavevector geometry (or the two-dimensional geometry) and the parallel wavevector geometry (or the slab geometry)

The first effect or geometry comes from the stiffness of the large-scale magnetic field. By analogy to a stiff string, the tension makes the magnetic field lines hard to bend, but the field lines (or strings) can be easily displaced in the plane perpendicular to the mean field. Turbulence can thus evolve in the perpendicular plane such that both the fluctuating fields and the wavevectors are confined in the perpendicular plane, e.g., eddies or perpendicular propagating fluctuations in the plane. This fluctuation component is referred to as the quasi-two-dimensional turbulence or the perpendicular wavevector geometry (Matthaeus et al. 1990; Biskamp 2003). The energy spectrum exhibits an extension or an elongation perpendicular to the mean magnetic field. Equivalently, spatial correlation is larger along the mean magnetic field, and shorter in the perpendicular direction, because only large-scale variations (or bending of the field lines) are possible along the magnetic field direction. Fluctuation geometry and the corresponding wavevector spectrum shape and the spatial correlation distribution are illustrated in Fig. 12 left panel.

The second effect of the mean magnetic field is the energy transport by the Alfvén waves in the parallel direction, as represented by waves or disturbance propagating along the string. Disturbance in the plasma and the magnetic field propagates as Alfvén waves along the mean field, as the Alfvén wave group velocity is either parallel or anti-parallel to the mean field. If plasma turbulence represents a set of field-aligned Alfvén wave packets, the energy spectrum shows an extension in the parallel and anti-parallel directions. Fluctuation geometry (also called the slab geometry) shows various wavelengths in the field-aligned direction. The spatial correlation is the longest in the perpendicular direction. (Fig. 12 right panel). The energy spectrum in the wavevector domain is modeled in an additive fashion for the slab and the two-dimensional fluctuation geometries in the two-dimensional wavevector domain (Bieber et al. 1996; Saur and Bieber 1999; Forman et al. 2011):

55 $$\begin{aligned} E(k_{\perp }, k_\Vert )= & {} E_\mathrm{sl}(k_{\perp }, k_\Vert ) + E_\mathrm{2d}(k_{\perp }, k_\Vert ) \end{aligned}$$

56 $$\begin{aligned} E_\mathrm{sl}= & {} C_\mathrm{sl} \left| k_\Vert \right| ^{-\alpha _\mathrm{sl}} \delta (k_{\perp }) \end{aligned}$$

57 $$\begin{aligned} E_\mathrm{2d}= & {} C_\mathrm{2d} \left( k_{\perp }^2 \right) ^{-(\alpha _\mathrm{2d}+1)/2} \delta (k_\Vert ) . \end{aligned}$$

Here the energy spectrum is constructed as power-law for the both geometries with different spectral indices.

*Perpendicular cascade scenario*

The picture of the perpendicular wavevector geometry is constructed by considering three-wave couplings for the Alfvén waves (Shebalin et al. 1983; Biskamp 2003). The three-wave resonance conditions in the frequencies $\omega$ and the wavevectors $\mathbf {k}$ are expressed in Eqs. (45) and (46), respectively. If one imposes that all the participating waves are the Alfvén waves with the dispersion relation $\omega = \mathbf {k} \cdot \mathbf {V}_\mathrm{A}$, the system of equations results in that the parallel wavevector component is zero for one interacting or participating wave, and is the same between the other participating wave and the generated wave such that $k_{\Vert (a)} = 0$ or $k_{\Vert (b)} = 0$. Since the frequency of the Alfvén wave is zero for the perpendicular propagation, one interacting wave component is a non-propagating spatial structure. The generated wave (wave component c) has a larger perpendicular wavevector component, and the cascade can proceed in the perpendicular direction. Two counter-propagating Alfvén waves as in the magnetohydrodynamic turbulence phenomenology by Iroshnikov (1964) and Kraichnan (1965a) cannot interact with each other in a three-wave coupling order, but at least in a four-wave coupling order or higher. If the cascade continues in the perpendicular direction, the wavelengths become smaller across the large-scale magnetic field while the wavelengths do not change in the parallel direction. The perpendicular cascade causes the formation of filament structures in plasma.

*Parallel cascade scenario*

Energy cascade is possible in the parallel direction under various conditions, for example, when four waves are even more waves are involved in the wave–wave interactions or when the conversion is possible into other wave modes such as the sound mode or dispersive modes. In the four-wave interactions, the wavevectors of the generated waves cannot be determined uniquely (and the parallel component of the wavevector is no longer constant). If the mode conversion is possible, a large-amplitude Alfvén wave collapses in a three-wave coupling sense into a forward-propagating sound wave (with respect to the direction of the original Alfvén wave) and a backward-propagating Alfvén wave, known as the decay or modulational instabilities (Derby 1978; Goldstein 1978). An Alfvén wave can also collapse into two forward-propagating daughter waves, (Mio et al. 1976; Mjølhus 1976; Nariyuki and Hada 2006). The generated waves do not have to lie on the dispersion relation. If the dispersion relation is not linear (straight line) but dispersive (curved line) and the propagation speed is frequency-dependent, the generated wave may happen to be on the dispersion relation. The decay and the modulational instabilities are systematically and numerically studied (Longtin and Sonnerup 1986; Terasawa et al. 1986; Wong and Goldstein 1986) in view of Hall-magnetohydrodynamics in which circular polarized large-amplitude Alfvén waves are obtained as an exact solution

One of the useful approximations is to regard turbulent fluctuations as a superposition of the fluctuation component for parallel-propagating waves (to the mean magnetic field, referred to as the slab geometry) and that for perpendicular wavevectors (referred to as the quasi-two-dimensional turbulence geometry). In solar wind turbulence, the fluctuation geometry for quasi-two-dimensional turbulence is estimated to have larger fluctuation amplitudes (Bieber et al. 1996). The dominance of quasi-two-dimensional turbulence is also indicated in the study of cosmic ray transport (a long mean-free-path of cosmic ray diffusion) (Bieber et al. 1994). On the other hand, the dominance of the slab or the quasi-two-dimensional fluctuation geometry can be case-dependent such that the low-speed stream in the solar wind is characterized by the quasi-two-dimensional turbulence geometry and the high-speed stream by the slab geometry (Dasso et al. 2005).

### Critical balance model

A different approach of constructing an anisotropic energy spectrum is to employ the critical balance hypothesis. Magnetohydrodynamic turbulence may be driven by eddies (which originate in fluid dynamics) and Alfvén waves (which originate in the electromagnetic field coupling with the plasma). One may phenomenologically construct a model by equating or regulating the two distinct time scales between the eddy turnover time and the Alfvén wave interaction time, referred to as the critical balance (Goldreich and Sridhar 1995),

58 $$\begin{aligned} \frac{v_\mathrm{ed}}{\ell _\perp } \sim \frac{V_\mathrm{A}}{\ell _\Vert } \,, \end{aligned}$$

where $v_\mathrm{ed}$ denotes the flow speed of the eddy around the direction of the large-scale magnetic field on the spatial scale perpendicular to the large-scale field $\ell _\perp$. The time scale of eddy splitting is constrained to that of the Alfvén wave passing time over the field-aligned spatial scale $\ell _\Vert$. In addition, we use Kolmogorov’s scaling for the energy transfer rate $\epsilon$ as

59 $$\begin{aligned} \epsilon \sim \frac{v_\mathrm{ed}}{\ell _\perp } . \end{aligned}$$

From Eqs. (58) and (59) we obtain the power-law relation between the parallel and the perpendicular components of the wavevectors [see, for example, derivation by Biskamp (2003)]

60 $$\begin{aligned} k_\Vert \propto \,\, k_0^{1/3} k_\perp ^{2/3} \end{aligned}$$

The critical balance hypothesis predicts that the energy cascade (as measured by the direction of the spectral extension in the wavevector domain) is not parallel nor perpendicular to the large-scale magnetic field, but the spectral extension becomes enhanced in the perpendicular direction to the mean magnetic field at larger wavenumbers.

The energy spectrum for the critical balance is modeled as (Forman et al. 2011)

61 $$\begin{aligned} E_\mathrm{cb}(k_{\perp }, k_\Vert ) = E_0 \left( \frac{k_\perp }{k_0} \right) ^{-10/3} g\left( - \left( \frac{k_\Vert }{k_0} \right) \left( \frac{k_\perp }{k_0} \right) ^{-2/3} \right) . \end{aligned}$$

**Fig. 13:**
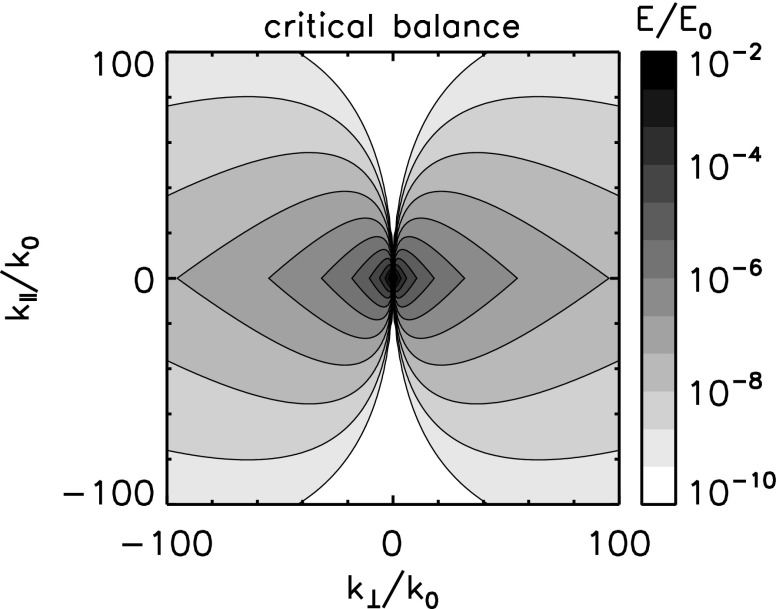
Energy spectrum in the wavevector domain for the critical balance model. The wavevector domain spans the perpendicular components (x-axis) and the parallel components (y-axis) to the mean magnetic field. The reference wavenumber $k_0$ is set to $k_0 = 10^{-5}\,\mathrm{rad \; km^{-1}}$. The spectrum is normalized to $E_0$ such that the integration of the spectrum over the wavevector domain becomes unity

Here the function *g*(*z*) satisfies the conditions $g(0)=1$, $\int _0^\infty g(z)\,\mathrm{d}z=1$, $\langle z \rangle = \int _0^\infty z g(z) \, \mathrm{d}z \sim 1$. For example, $g(z)=\exp \left[ -z \right]$ is one possible choice. One-dimensional spectra are power-law, and obtained as $E(k_\perp ) \propto k_\perp ^{-5/3}$ and $E(k_\Vert ) \propto k_\Vert ^{-2}$, respectively. An example of the energy spectrum in the wavevector domain for the critical balance hypothesis is displayed in Fig. 13 for the exponential function of *g*(*z*).

The critical balance hypothesis successfully explains the change in the spectral slope as a function of the projection angle from the mean magnetic field (Forman et al. 2011). The scaling relation is found to be valid in numerical simulations (Cho and Vishnian 2000) and the relation between the wavevector components is also confirmed in the inner heliosphere (He et al. 2013).

### Elliptic anisotropy model

Numerical simulation of MHD turbulence also supports the anisotropic energy spectrum. A nearly elliptic shape of the energy spectrum is obtained from magnetohydrodynamic turbulence simulation (Shebalin et al. 1983) in which the initial spectrum is set to isotropic, and anisotropy develops in such a way that the spectrum extends perpendicular to the large-scale magnetic field forming an elliptic shape. Elliptic sense of the wavevector anisotropy is the simplest and a natural extension of the energy spectrum from the isotropic case to an anisotropic one. The reason for this lies in the fact that the elliptical shape appears in the lowest-order (second-order) polynomial expansion of a smooth function in the two-dimensional domain such as a space–time correlation function (He and Zhang 2006).

The elliptic wavevector anisotropy represents a set of elliptic contours of the energy spectrum in the wavevector domain (Bruno and Carbone 2013; Carbone et al. 1995; Narita 2014). The energy spectrum in the two-dimensional wavevector domain spanning the parallel and perpendicular components to the mean magnetic field is modeled as

62 $$\begin{aligned} E(k_\perp , k_\Vert ) = E_0 \left( c_\perp k_\perp ^2 + c_\Vert k_\Vert ^2 \right) ^{-\alpha /2} \,. \end{aligned}$$

The following parameters are needed to construct an elliptically anisotropic spectrum: the shape parameters (denoted by $c_\perp$ and $c_\Vert$) and the power-law index (denoted by $-\alpha$). The spectral index $-\alpha$ can reproduce an index of 5 / 3, the one-dimensional Kolmogorov inertial range spectrum, in the isotropic limit ($c_\perp / c_\Vert \rightarrow 1$). To simplify the argument, the other coefficients in the inertial-range spectrum such as the Kolmogorov or Iroshnikov–Kraichnan constant (Kolmogorov 1991; Iroshnikov 1964; Kraichnan 1965a) and the energy transfer rate $\epsilon$ are renormalized to $E_0$ in Eq. (62).

**Fig. 14:**
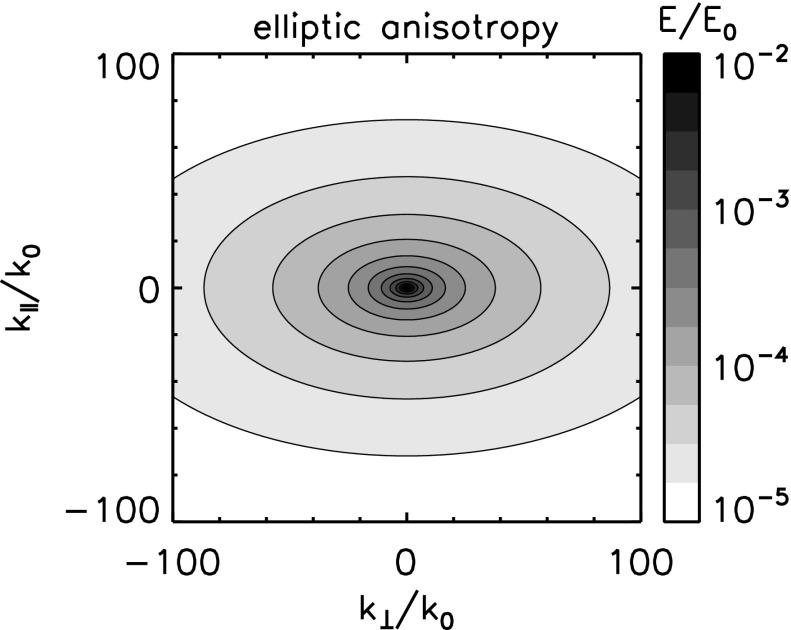
Energy spectrum in the wavevector domain for the elliptic model. The coordinates and the normalizations are the same as that in Fig. 13. The elliptic shape is set to a coefficient of $c=0.3$

The sense of elliptic shape does not change over the scales or the wavenumbers (Fig. 14). The one-dimensional spectra are expressed as a power law in the elliptic anisotropy model, and the spectral index is the same between the parallel and perpendicular directions to the mean magnetic field. The isotropic spectrum is restored by taking the limit $c_\perp /c_\Vert \rightarrow 1$, and Kolmogorov’s inertial range spectrum is restored by comparing the spectral index of the one-dimensional spectrum as $-\alpha + 1 = -5/3$. Therefore, anisotropy cannot be measured solely from the analysis of the spectral indices lone because the effect of anisotropy appears in the coefficients, not in the spectral slope. To verify the elliptic anisotropy using one-dimensional spectra, one needs to measure the spectra both in the perpendicular and parallel directions to the large-scale magnetic field simultaneously. One-dimensional spectra are obtained by integrating the elliptic spectrum over the wavevector components:

63 $$\begin{aligned} E(k_\perp )= & {} E_0 \int _{-\infty }^\infty \mathrm{d}k_\Vert \, E(k_\perp , k_\Vert ) = E_0 C_\perp k_\perp ^{-\alpha +1} \end{aligned}$$

64 $$\begin{aligned} E(k_\Vert )= & {} E_0 \int _{-\infty }^\infty \mathrm{d}k_\perp \, E(k_\perp , k_\Vert ) = E_0 C_\Vert k_\Vert ^{-\alpha +1} \,, \end{aligned}$$

where $C_\perp$ and $C_\Vert$ denote the spectral amplification factors that determine the spectral energy in the one-dimensional wavenumber domain in different directions with respect to the mean magnetic field. The coefficients $C_\perp$ and $C_\Vert$ are evaluated using Euler’s gamma function as

65 $$\begin{aligned} C_\perp= & {} 2 c_\perp ^{-\alpha /2} \int _0^\infty \mathrm{d}\xi \, \left( 1 + \frac{c_\Vert }{c_\perp } \xi ^2 \right) ^{-\alpha /2} \nonumber \\= & {} 2 c_\perp ^{-\alpha /2} \sqrt{\frac{c_\perp \pi }{c_\Vert }} \frac{\varGamma (-\frac{1}{2}+\frac{\alpha }{2})}{\varGamma (\frac{\alpha }{2})} \,. \end{aligned}$$

The coefficient $C_\Vert$ is obtained by replacing $c_\perp$ by $c_\Vert$ and vice versa. The elliptic spectrum assumes a symmetry with respect to changing the sign of the wavevector components along the mean magnetic field, i.e., the cross helicity is implicitly zero. The scale invariance holds under the transformation $k_\perp \rightarrow \lambda k_\perp$ and $k_\Vert \rightarrow \lambda k_\Vert$.

### Non-elliptic anisotropy model

The spectral index of the frequency spectra in the solar wind depends on the angle of the flow (streamwise direction) from the large-scale magnetic field (Horbury et al. 2008; Osman and Horbury 2009), $-\,5/3$ for the quasi-perpendicular projection and $-\,2$ for the quasi-parallel projection. The explanation of the change in the spectral index can not only be explained by the critical balance hypothesis but also by a variation of the elliptic spectrum into a non-elliptic spectral shape. The energy spectrum for non-elliptic wavevector anisotropy is modeled as

66 $$\begin{aligned} E(k_\perp , k_\Vert ) = E_0 \left( c_\perp \left( \frac{k_\perp }{k_0} \right) ^2 + c_\Vert \left( \frac{|k_\Vert |}{k_0} \right) ^3 \right) ^{-7/6} \,. \end{aligned}$$

Equation (66) is obtained by imposing $E(k_\perp ) \propto k_\perp ^{-5/3}$ and $E(k_\Vert ) \propto k_\Vert ^{-2}$ on a generalized form of the elliptic anisotropy, $(c_\perp k_\perp ^2 + c_\Vert |k_\Vert |^\mu )^{-\alpha /2}$ (Narita 2015). The idea of non-elliptic anisotropy is driven by the observational facts that the perpendicular and parallel spectra (in the one-dimensional wavenumber domain) have different spectral indices and that the multi-spacecraft observations show a change of the spectral extension direction from the parallel direction on large scales into the perpendicular direction on smaller scales. Integration of the two-dimensional spectrum over the wavevector components orthogonal to the direction of interest yields one-dimensional spectra:

67 $$\begin{aligned} E(k_\perp )= & {} \int _{-\infty }^{\infty } \, \mathrm {d}k_\Vert \, E(k_\perp , k_\Vert ) \end{aligned}$$

68 $$\begin{aligned}= & {} E_0 \int _{-\infty }^{\infty } \, \mathrm {d}k_\Vert \, (c_\perp k_\perp ^2 + c_\Vert |k_\Vert |^\mu )^{-\alpha /2} \end{aligned}$$

69 $$\begin{aligned}\propto & {} k_\perp ^{-\alpha +\frac{2}{\mu }} \end{aligned}$$

70 $$\begin{aligned} E(k_\Vert )= & {} \int _{-\infty }^{\infty } \, \mathrm {d}k_\perp \, E(k_\perp , k_\Vert ) \end{aligned}$$

71 $$\begin{aligned}= & {} E_0 \int _{-\infty }^{\infty } \, \mathrm {d}k_\perp \, (c_\perp k_\perp ^2 + c_\Vert |k_\Vert |^\mu )^{-\alpha /2} \end{aligned}$$

72 $$\begin{aligned}\propto & {} k_\Vert ^{-\frac{\mu }{2}(\alpha - 1)} \end{aligned}$$

The power-law indices $\mu$ and $\alpha$ are obtained algebraically as $\mu = 3$ and $\alpha = 7/3$ by solving the following equations:

73 $$\begin{aligned} -\alpha +\frac{2}{\mu }= & {} -\frac{5}{3} \end{aligned}$$

74 $$\begin{aligned} -\frac{\mu }{2}(\alpha - 1)= & {} -2 \,. \end{aligned}$$

**Fig. 15:**
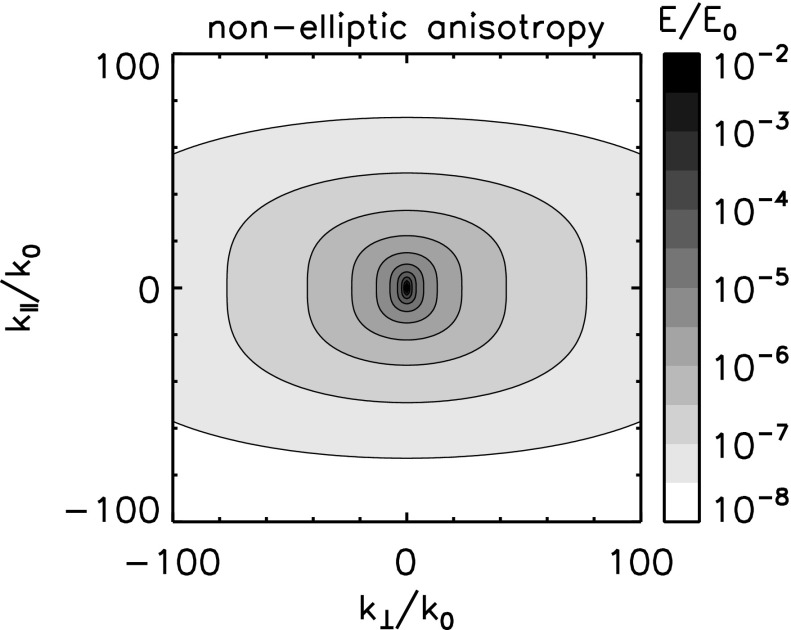
Energy spectrum in the wavevector domain for the non-elliptic model. The coordinates and the normalizations are the same as that in Fig. 13

More complete forms of the one-dimensional spectra are

75 $$\begin{aligned} E(k_\perp )= & {} E_0 \, C_{\perp } \, k_\perp ^{-5/3} \end{aligned}$$

76 $$\begin{aligned} E(k_\Vert )= & {} E_0 \, C_\Vert \, k_\Vert ^{-2} \,, \end{aligned}$$

where the coefficients $C_{\perp }$ and $C_{\Vert }$ are the spectral amplification factors obtained by the integrations in Eqs. (68) and (71) as

77 $$\begin{aligned} C_\perp= & {} 2 c_\perp ^{-\alpha } \left( \frac{c_\perp }{c_\Vert } \right) ^{1/\mu } \frac{\varGamma \left( \frac{\alpha }{2} - \frac{1}{\mu } \right) \varGamma \left( 1 + \frac{1}{\mu } \right) }{\varGamma \left( \frac{\alpha }{2} \right) } \end{aligned}$$

78 $$\begin{aligned} C_\Vert= & {} c_\Vert ^{-\alpha /2} \sqrt{\frac{c_\Vert \pi }{c_\perp }} \frac{\varGamma \left( -\frac{1}{2} + \frac{\alpha }{2} \right) }{\varGamma \left( \frac{\alpha }{2} \right) } , \end{aligned}$$

and by setting $\mu =3$ and $\alpha = 7/3$. Here, the gamma function $\varGamma (x)$ is introduced in Eqs. (77) and (78). An example of the non-elliptic wavevector anisotropy is displayed in Fig. 15. The sense of the wavevector anisotropy changes gradually from a spectral extension in the parallel direction (to the mean magnetic field) on larger scales or at smaller wavenumbers into an extension in the perpendicular direction on smaller scales or at larger wavenumbers.

### Asymmetries

The energy spectrum may appear asymmetrically in two different ways with respect to the direction of the mean magnetic field. One is the energy imbalance between the parallel and the anti-parallel direction to the mean field, and the other is an asymmetry in the azimuthal directions around the mean field.

The energy imbalance between the parallel and the anti-parallel directions is a realization of a finite cross helicity, $h_\mathrm{c} = \langle \mathbf {U} \cdot \mathbf {B}/\sqrt{\mu _0 \rho _0} \rangle = \langle |z^+|^2 \rangle - \langle |z^-|^2 \rangle$, where $z^{\pm } = \mathbf {U} \pm \frac{\mathbf {B}}{\sqrt{\mu _0 \rho _0}}$ are the Elsässer variables representing the anti-parallel and parallel propagating Alfvén waves (to the mean magnetic field) for the plus and the minus signs, respectively. Large-scale or magnetohydrodynamic turbulent fluctuations in the inner heliosphere exhibit finite values of the cross helicity (Tu and Marsch 1995). The one-dimensional energy spectrum in the perpendicular wavenumber domain under a finite cross helicity is modeled phenomenologically by correcting the Kolmogorov-type spectrum (with a spectral index of $-5/3$) for the normalized cross helicity $\sigma _\mathrm{c}$ as (Chandran 2008):

79 $$\begin{aligned} E(k_\perp )\propto & {} k_\perp ^{-\alpha } \end{aligned}$$

80 $$\begin{aligned} \alpha= & {} \frac{5}{3} + \frac{2}{3} \frac{ \log _{10} \left( \frac{ 1+\sigma _\mathrm{c} }{ 1-\sigma _\mathrm{c} } \right) }{ \log _{10} \left( \frac{ k_\mathrm{d} }{ k_\mathrm{f} } \right) } \end{aligned}$$

81 $$\begin{aligned} \sigma _\mathrm{c}= & {} \left( \frac{ \left| z^+ \right| ^2 - \left| z^- \right| ^2 }{ \left| z^+ \right| ^2 + \left| z^- \right| ^2 } \right) _{k_\mathrm{f}} \,, \end{aligned}$$

where $k_\mathrm{d}$ and $k_\mathrm{f}$ denote the dissipation-scale wavenumber (a large wavenumber) and the forcing-scale or energy-containing-scale wavenumber (a small wavenumber). The normalized cross helicity is measured at the forcing-scale wavenumber. The spectral index varies from $-5/3$ for $\sigma _\mathrm{c}=0$ (energy-balanced case) to nearly $-2$ for $\sigma _\mathrm{c} \simeq \pm 1$ (energy-unbalanced case).

Axial asymmetry is indicated by in situ measurements in the solar wind. Using single spacecraft data and a mapping procedure, the three-dimensional structure of solar wind turbulence is obtained from the Ulysses spacecraft during a polar pass at the heliocentric distance 1.4–2.6 AU in 1995 (Chen et al. 2012). The fluctuations are axially asymmetric in the directions around the mean magnetic field. Using multi-spacecraft data, the axially asymmetric energy spectrum is presented directly in the three-dimensional wavevector domain (Narita 2014). At the time of the manuscript writing, there is no direct clue as to the origin or the mechanism of the axial asymmetry. The mechanism may stem from an asymmetric radial flow expansion in the heliosphere or an intrinsic plasma process.

### Lessons from the observations

Observationally speaking, it is possible to estimate, using some assumptions, the energy spectrum in the wavevector domain by two different mapping methods from the frequency domain onto the wavevector domain.

**Fig. 16:**
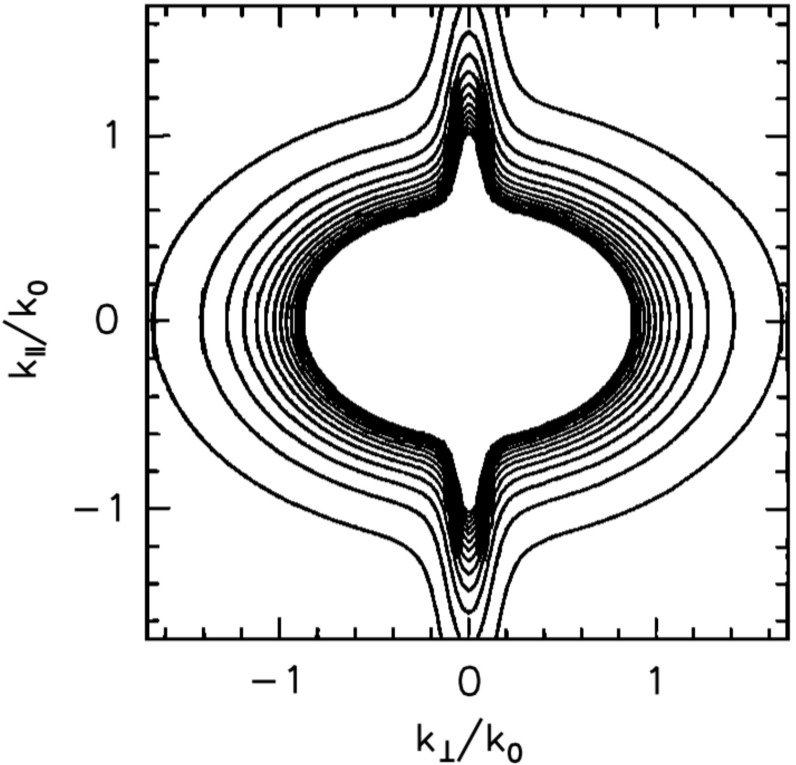
Contours of the energy spectrum in the wavevector domain estimated from the single spacecraft measurements in the solar wind. Image reproduced with permission from Carbone et al. (1995), copyright by AGU

The first mapping method is the Fourier transform of the spatial correlations onto the wavevectors. Under the assumptions of (1) statistical homogeneity, (2) time stationarity, (3) divergence-free condition of the magnetic field, (4) Taylor’s frozen-in flow hypothesis, and (5) spatial symmetries with respect to a reflection by changing the sign of the wavevector ($\mathbf {k} \rightarrow -\mathbf {k}$) and a reflection by changing the sign of the parallel component of the wavevector ($k_\Vert \rightarrow -k_\Vert$), the correlation tensor and the spectra are determined by fitting a model correlation tensor against the spacecraft data and obtaining the parameter set for the model correlation tensor. Figure 16 displays the contour lines of the energy spectrum obtained by that procedure (Carbone et al. 1995) for a time interval of Alfvénic fluctuations measured by the Helios-2 magnetic field data in the inner heliosphere. The spectrum exhibits two populations, one extending parallel or anti-parallel to the mean magnetic field, and the other extending perpendicular to the mean field. The energy spectra are also determined by He et al. (2013) directly in the wavevector domain again, by assuming Taylor’s hypothesis and by mapping the time correlation of the frequency spectrum onto the wavevector domain, showing a supporting evidence for the critical balance hypothesis with a primary spectral extension direction highly oblique from the mean magnetic field.

The second mapping method assumes the existence of the dispersion relations and the associated polarizations of the field fluctuations, in contrast to the use of Taylor’ hypothesis $\omega _\mathrm {sc} = \mathbf {k}\cdot \mathbf {U}$ using the flow velocity $\mathbf {U}$. The concept of the wave distribution function has been developed to project the measured covariance matrix in the frequency domain onto the wave energy in the wavevector domain using a given set of the dispersion relations $\omega _0(\mathbf {k})$ and the associated polarization matrix in the wavevector domain (Storey and Lefeuvre 1979, 1980; Lefeuvre et al. 1982; Oscarsson and Rönnmark 1989, 1990; Oscarsson 1994; Oscarsson et al. 2001; Santolik and Parrot 1996) Mathematically, the projection is an inverse problem of estimating the wave energy in the wavevector domain $F(\mathbf {k})$ from the measured covariance matrix $R_{ij}$ in the frequency domain by solving or optimizing the following equation:

82 $$\begin{aligned} R_{ij}(\omega _\mathrm {sc}) = \int \, \mathrm {d}^3k \, \mathbf {a}_{ij}(\mathbf {k})\, F(\mathbf {k}) \, \delta \left( \omega _\mathrm {sc} - \omega _0(\mathbf {k}) \right) , \end{aligned}$$

where $\mathbf {a}_{ij}(\mathbf {k})$ denotes the polarization matrix for a given wavevector and the dispersion relation $\omega _0(\mathbf {k}0)$ is imposed by the Dirac delta function.

**Fig. 17:**
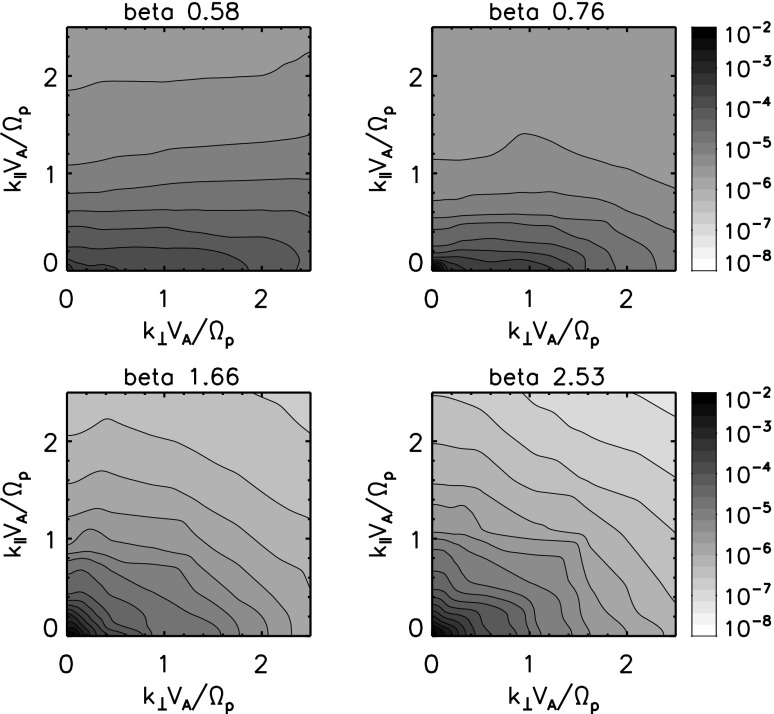
Energy spectra in the wavevector domain for magnetic field fluctuations in the solar wind obtained from multi-point measurements using the Cluster spacecraft under various conditions of ion beta values. Image reproduced with permission from Narita et al. (2014), copyright by the author

Wavevector anisotropies can be determined directly in the wavevector domain using multi-spacecraft data. Advantages are on the direct measurements without using Taylor’s hypothesis or assuming the dispersion relation. Figure 17 displays the magnetic energy spectra in the solar wind in the domain of parallel and perpendicular wavevector components to the mean magnetic field. The spectra are determined in the four-dimensional Fourier domain spanning the frequencies (in the spacecraft frame) and the three components of wavevectors, and reduced to two-dimensional spectra by integrating over the frequencies, averaging over the directions around the mean magnetic field, and folding over the sign of the parallel component of the wavevectors. The wavevector anisotropy is stronger at lower values of beta, and weaker (i.e., more isotropic) at higher values of beta. Overall, the primary extention direction of the anisotropic energy spectra is perpendicular to the mean magnetic field.

## Outlook

Turbulence evolves both spatially and temporally while retaining or restoring intrinsic scaling symmetry. While turbulent fluctuations may appear nearly random in the spatial coordinates and in the time series data, the energy spectrum in the wavevector-frequency cannot be random but exhibits a particular spectral shape, reflecting the propagation nature and the energy cascade directions. An analysis or a study of the energy spectra in the wavevector-frequency domain enables us to obtain a more comprehensive or integrated picture of turbulence, including the following aspects:

- applications and limits of Taylor’s hypothesis
- Doppler shift and Doppler broadening
- existence of dispersion relations
- energy cascade directions
- visualization of of anisotropies and asymmetries

All these items can be discussed as realizations or different slices of the energy spectrum. Revisiting Taylor’s hypothesis from higher dimensions leads us naturally to the notion of dispersion relations. In plasma turbulence, various kinds of dispersion relations exist as plasma dynamics is necessarily coupled to the electromagnetic field. Dispersion relations are dependent on the directions of the wavevectors, and the wavevector anisotropy is closely coupled to the existence of the dispersion relations. Doppler shift and broadening, dispersion relations, wavevector anisotropies, and axial asymmetries are visualized by slicing the energy spectrum at various frequencies or wavevectors or by integrating the spectrum over the frequencies or the wavevectors. To conclude the review, we discuss some of future directions to maximize the scientific potential of the space–time structure study of plasma turbulence.

*Control parameters*

While the plasma parameter beta is often used as a control parameter in (roughly) determining the behavior of plasma dynamics, e.g., dispersion relations, there may be more control parameters. The fluctuation amplitude with respect to the mean field (for example, magnetic field) may be regarded as an index for the strength of nonlinearity. In fact, the notion of linear modes is valid only when the fluctuations are sufficiently small such that the wave–wave interactions are negligible. In reality, many waves including linear mode waves and nonlinear or sideband waves can be excited in plasma turbulence.

*Transition into fully-developed turbulence*

The frequency broadening around the Doppler shift (which appears as the Doppler broadening) and the dispersion relations (which appears as the sideband waves) has different possible origins and may plan an important role in turbulence evolution. While the fluctuation of the large-scale flow (or the random sweeping effect) causes the Doppler broadening, wave–wave coupling causes the sideband waves around the normal mode dispersion relations. Whether the waves generated by the three-wave coupling are supported by the normal mode can be studied by the mismatch test as in Gary (2013). One may, for example, regard the sideband waves as a proxy of turbulence evolution degree (how much the fluctuations are stored on the normal modes and the sideband waves) as in Comişel et al. (2013). The three-wave coupling model is illustrative and in that the model can combine different components such as the linear mode waves, the sideband waves, and the wavevector anisotropies. The lifetime of the sideband waves should be measured or studied in more detail to reveal the transition into turbulence.

*Mechanism causing axial asymmetry*

The axial asymmetry remains one of the unsolved problems in plasma turbulence. Both single spacecraft and multi-spacecraft measurements show evidence for the axial asymmetry. Naively speaking, there are two causes, one by intrinsic processes of plasma turbulence and the other by external processes such as an inhomogeneous flow or an spatially expanding flow. In the linear Vlasov theory treatment, axial symmetry is explicitly broken by selecting the direction of wave propagation (or the wavevector direction) and the coordinate system is constructed using the direction of the large-scale magnetic field and the direction of the wave propagation. Axial symmetry is already broken when deriving the normal mode dispersion relation. In the former scenario, since the fundamental equations are invariant under the rotation around the magnetic field direction, it is natural to anticipate the Nambu–Goldstone mode that compensates for the broken symmetry by exciting an oscillation in the sense of the original symmetry. This argument leads us to predict that the wavevector anisotropy might rotate in the temporal sense to compensate the broken axial asymmetry. In the latter scenario, the asymmetry is interpreted as caused by the large-scale flow effect, e.g., the expansion of the solar wind plasma from corona to the heliosphere stretches the wavelengths of turbulence in the radial direction from the sun, while the expansion does not stretch the wavelengths perpendicular to the radial direction.

*Co-existence with eddies*

The critical balance hypothesis postulates that both Alfvén waves and eddies regulate each other and imply that the both fluctuation types co-exist at the same time. It is an important task to study the existence of eddies around the large-scale magnetic field direction. Also, the three-dimensional picture of plasma turbulence may essentially be different from the two-dimensional one due to the additional degree of freedom in the directions around the mean magnetic field. Comparison in direct numerical simulations between two-dimensional and three-dimensional spatial settings would give a hint on this question.

*Spectral tensor*

We have treated the energy of the fluctuating field simply as a scalar *E*, but the fluctuations are found in the plasma quantities (density, flow velocity, temperature), the electric field, and the magnetic field. Furthermore, the fluctuation energy can be analyzed with respect to the field magnitude as well as to the components for the vectorial quantity, e.g., fluctuation parallel or perpendicular to the large-magnetic field. Since the energy spectrum is obtained as the Fourier transform of the correlation function, the analysis of the spectral energy is extended to the analysis of the spectral tensor in the wavevector-frequency domain. We here give an overview of the spectral tensor analysis for plasma turbulence.

*Compressible and incompressible sense*

The energy spectrum for the vectorial field is obtained by computing the cross spectral density matrix. Each element of the matrix represents essentially a correlations between different components of the fluctuating field (off-diagonal elements) or for the same component (diagonal elements). One may then compute the trace of the matrix and use it as the total fluctuation energy, as the trace is invariant under the coordinate system rotation. The diagonal and the off-diagonal elements of the tensor can be used in the data analysis by choosing a reasonable coordinate system. For example, the diagonal elements of the matrix in the mean-field-aligned coordinate system (oriented to the large-scale magnetic field direction) are the measure of the compressible (parallel to the large-scale field direction) and the incompressible sense (perpendicular to the large-scale field) of the fluctuating field. Another choice of the coordinate system is the eigenvector system, oriented to the principal axes of the fluctuations. If the fluctuating field is divergence-free, the direction of the minimum fluctuating field can be used as a measure of the wavevector direction with the 180-degree ambiguity (because one can determine only the normal direction to the plane of circular or elliptic polarization). Velocity fluctuations longitudinal and transverse to the wavevectors can be evaluated in the tensor analysis.

*Field rotation sense*

One may also use the off-diagonal elements of the spectral density matrix. These elements contain the information of the field rotation sense. The sense of the field rotation and the eccentricity of elliptically polarizing field can be obtained from the off-diagonal elements (temporal polarization). One may also compute the spatial helical sense of the fluctuation, and the helicity spectrum can be obtained directly in the wavevector domain using multi-spacecraft measurements. Not only energy but also helicity plays an important role in plasma turbulence, as in a closed system (bounded by a magnetic surface) both the total energy and the total helicity are the conserved quantities in ideal magnetohydrodynamics. Helical sense of the field can be measured for the magnetic field (magnetic helicity) and the flow velocity (kinetic helicity). Also, the energy spectrum can be computed for the right-hand and left-hand circularly polarized waves separately, which is a useful tool to study the detailed processes of wave–wave interactions.

*Correlation between plasma and magnetic field*

Correlation between the flow velocity and the magnetic field can be used to study the cross helicity in plasma turbulence, which is a measure of the difference between Alfvén waves propagating forward to the large-scale magnetic field and those propagating backward in incompressible magnetohydrodynamics. However, in the kinetic regime, the concept of the flow velocity breaks down, and one must look in detail at the fluctuation or the disturbance of the velocity distribution function. The use of the energy spectrum for the electric and the magnetic fields is valid in the kinetic regime but the energy spectrum may be different between the electric and the magnetic field, depending on the dispersion effect of the fluctuations. Also, the residual energy between the kinetic and the magnetic ones serves as a useful diagnostics tool in plasma turbulence.

The final process of turbulence (at the highest wavenumbers) after the energy cascade is the energy dissipation. The fluctuation energy is converted into the thermal energy. Different mechanisms exist in wave–particle interactions. The energy is transferred into the thermal one, for example, through the cyclotron resonance between waves and particles by means of the wave electric field perpendicular to the large-scale magnetic field or the Landau resonance by means of the parallel electric field. One may also track the time evolution of the thermal energy in plasma turbulence to study how much the medium is heated by turbulence.

*Phase information*

Turbulence is often associated with the random motion of the fluid. Naively speaking, one may anticipate that the fluctuation statistics exhibits the Gaussian distribution. In fact, we have also used the Gaussian frequency distribution to model the energy spectrum in the wavenumber–frequency domain. Fluctuations following the Gaussian distribution are called self-similar, that is, the statistics is scalable to different spatial or time lengths, and the fluctuations on all the possible length scales fill the space (which gives the notion of self-similarity).

Turbulence theories, however, predict that the fluctuations statistics should not be strictly Gaussian. One of the important properties of the Gaussian distribution is that the phases of the Fourier transformed fields are randomly distributed. If the distribution were Gaussian, waves comprising the fluctuations are completely incoherent and uncorrelated to each other. For completely random-phase fluctuations, there is no energy transport in the spectral domain, and the picture of the energy cascade in the inertial range breaks down. Deviation from the Gaussian statistics can be found in many physical systems. Fluctuations in the spatial coordinate or in the time series data often exhibit sparsely localized structures or spiky signals, respectively. In other words, fluctuations are not self-similar in that the picture of the spatial-filling pattern on all the scales is no longer valid. In fact, in a real turbulent flow, small-scale eddies or fluctuations become increasingly sparse, or *intermittent*, which is a sign of broken self-similarity. Realization of intermittency can be found in coherent wave–wave interactions (that are the main driver of the energy transport in the inertial range) and formation of coherent structures such as current sheets in plasmas. The appearance or the degree of intermittency can be studied using the probability density function (PDF) of the fluctuations. Or one may compute higher-order moments or cumulants of the PDFs and associate the deviation from the Gaussian distribution with the degree of intermittency. Multiple wave couplings can be studied using the method of higher-order correlations. Three-field correlation is called the bispectrum and it is a measure of phase coherence or strength of three-wave coupling. Likewise, one may compute four-field correlation (called the tri-spectrum) which is a measure of four-wave coupling. Multi-spacecraft measurements in space can be used for the studies of multiple wave couplings in the wavevector-frequency domain. The extension of the four-dimensional energy spectrum to the tensorial treatment and the higher-order moments will give a more complete picture of plasma turbulence in space and astrophysical systems.
